# Functional Identification of Serine Hydroxymethyltransferase as a Key Gene Involved in Lysostaphin Resistance and Virulence Potential of *Staphylococcus aureus* Strains

**DOI:** 10.3390/ijms21239135

**Published:** 2020-11-30

**Authors:** Nayab Batool, Kwan Soo Ko, Akhilesh Kumar Chaurasia, Kyeong Kyu Kim

**Affiliations:** 1 Department of Precision Medicine, Institute for Antimicrobial Resistance Research and Therapeutics, Sungkyunkwan University School of Medicine, Suwon 16419, Korea; nayyab114@gmail.com; 2 Department of Microbiology, Sungkyunkwan University School of Medicine, Suwon 16419, Korea; ksko@skku.edu; 3 Samsung Advanced Institute for Health Sciences and Technology (SAIHST), Samsung Medical Center (SMC), Sungkyunkwan University School of Medicine, Seoul 06351, Korea

**Keywords:** *Staphylococcus aureus*, ST72, lysostaphin resistance, folate cycle, serine hydroxymethyltransferase, SHMT, virulence factor, SHMT inhibitor

## Abstract

Gaining an insight into the mechanism underlying antimicrobial-resistance development in *Staphylococcus aureus* is crucial for identifying effective antimicrobials. We isolated *S. aureus* sequence type 72 from a patient in whom the *S. aureus* infection was highly resistant to various antibiotics and lysostaphin, but no known resistance mechanisms could explain the mechanism of lysostaphin resistance. Genome-sequencing followed by subtractive and functional genomics revealed that serine hydroxymethyltransferase (*glyA* or *shmT* gene) plays a key role in lysostaphin resistance. Serine hydroxymethyltransferase (SHMT) is indispensable for the one-carbon metabolism of serine/glycine interconversion and is linked to folate metabolism. Functional studies revealed the involvement of SHMT in lysostaphin resistance, as Δ*shmT* was susceptible to the lysostaphin, while complementation of the knockout expressing *shmT* restored resistance against lysostaphin. In addition, the Δ*shmT* showed reduced virulence under in vitro (mammalian cell lines infection) and in vivo (wax-worm infection) models. The SHMT inhibitor, serine hydroxymethyltransferase inhibitor 1 (SHIN1), protected the 50% of the wax-worm infected with wild type *S. aureus*. These results suggest SHMT is relevant to the extreme susceptibility to lysostaphin and the host immune system. Thus, the current study established that SHMT plays a key role in lysostaphin resistance development and in determining the virulence potential of multiple drug-resistant *S. aureus*.

## 1. Introduction

Exposure of bacterial pathogens to antibiotic stress, resulting in the clonal selection of antibiotic-resistant bacterial pathogens, poses a threat to human society and healthcare systems [1,2]. Among the various antimicrobial resistance (AMR) bacterial pathogens, *Staphylococcus aureus* is one of the leading causes of diseases ranging from skin and soft tissue infections (SSTI) to lethal sepsis, pneumonia, and endocarditis [3,4,5]. The evolution of AMR has been driven by high metabolic adaptability [6], acquisition of AMR genes by conjugal transfer and their stable inheritance either as plasmid or as cis-elements [7], and phage-integration [8] to mutations in the chromosome of *S. aureus*, which are continuously being clonally enriched in response to exposure to various antibiotics [9]. Various studies have revealed the presence of multiple sequence types in community and hospital-associated methicillin-resistant *S. aureus* (MRSA) strains worldwide, including in South Korea [10]. Among the major sequence types (ST), ST72 was found to be the leading pathogen colonizing the nares in all age groups of patients suffering from *S. aureus* infections in South Korea [11]. The ever-increasing AMR clones of bacterial pathogens have prompted scientists to develop alternative therapies [12,13,14] against novel potential antimicrobial targets to combat the multiple drug resistant (MDR) infections in community as well as hospital settings [15].

An observational pediatric study in South Korea (2014–2015) revealed ST72-staphylococcal cassette chromosome mec (SCC*mecIV*) to be the major colonizing and pathogenic genotype which accounts for nearly 32% of clinical MRSA infections [16]. ST72 isolates showed differential levels of resistance against several antibiotics, including vancomycin [15]. Besides antibiotics, lysostaphin, a bacteriocin, is one of the most effective and specific anti-staphylococcal enzymes secreted by *Staphylococcus simulans* biovar *staphylolyticus* [17,18]. It is a zinc-metalloendopeptidase that cleaves the pentaglycine bridge in the peptidoglycan layer of the cell wall [19], resulting in the loss of cellular integrity [20]. Lysostaphin is an autolysin that contains a cell wall binding domain and catalytic cleavage domain [21,22]. The use of lysostaphin is more advantageous than that of antibiotic regimes, by virtue of certain properties of lysostaphin such as specificity for staphylococci and extremely high-killing kinetics [23]. Lysostaphin is highly effective against methicillin-resistant/susceptible *S. aureus* (MRSA/MSSA) [24], vancomycin-resistant, and vancomycin intermediate-resistant *S. aureus* [25] at all stages of bacterial growth [26]. 

The current study assessed the lysostaphin-mediated killing of 11 MRSA and MSSA ST72 isolates isolated from human, animal, and soil samples in South Korea [27]. We found that K07-204, a human isolate of ST72, displayed resistance against lysostaphin, which was further confirmed by the colony forming unit (CFU) assay and confocal and scanning electron microscopy; based on these analyses, this strain was identified as lysostaphin-resistant isolate (*lys^r^*) of ST72. To elucidate the resistance mechanism, all known genes and mutations associated with lysostaphin resistance were cloned and screened in lysostaphin-resistant (*lys^r^*, K07-204) versus susceptible (*lys^s^*, K07-561) ST72 human isolates, including the endopeptidase resistance gene (*epr*), *femABX* family [*femA*, *femB*, *femX* (*fmhB*)], and homologs of the *epr* gene (*eprh* or *fmhC*). Surprisingly, the sequences of all the aforementioned genes were found to be identical to the known sequences [28,29] in lysostaphin-susceptible strains, suggesting that none of the known mechanisms of lysostaphin resistance exist in K07-204. Therefore, we employed a comparative and functional genomics approach for identifying unknown mechanisms of lysostaphin resistance. Based on the results of metabolic pathway modeling using whole genome sequence, followed by comparative genomics, we hypothesized the role of serine hydroxymethyl transferase (*glyA,* hereafter referred to as ‘*shmT’* for gene and ‘SHMT’ for enzyme) in the development of lysostaphin resistance. We confirmed its relevance by showing that the *shmT* knockout showed susceptibility to lysostaphin, while its ectopic overexpression driven by the tetracycline-inducible promoter paradoxically causes extreme susceptibility to lysostaphin. The use of a specific inhibitor of SHMT, SHIN1 (6-Amino-1,4-dihydro-4-[5-(hydroxymethyl)[1,1′-biphenyl]-3-yl]-3-methyl-4-(1-methylethyl)pyrano [2,3-c]pyrazole-5-carbonitrile), revealed that *S. aureus* USA300 was found to be highly susceptible to the host immune system. Thus, we established the role of SHMT in the lysostaphin resistance development and in determining the virulence potential of *S. aureus*. Collectively, the current study demonstrates that SHMT contributes towards lysostaphin resistance/susceptibility and acts as a potent virulence factor for increasing the susceptibility of MDR *S. aureu*s strains to host.

## 2. Results

### 2.1. Lysostaphin Resistance Pattern in ST72 Isolates

**Figure 1:**
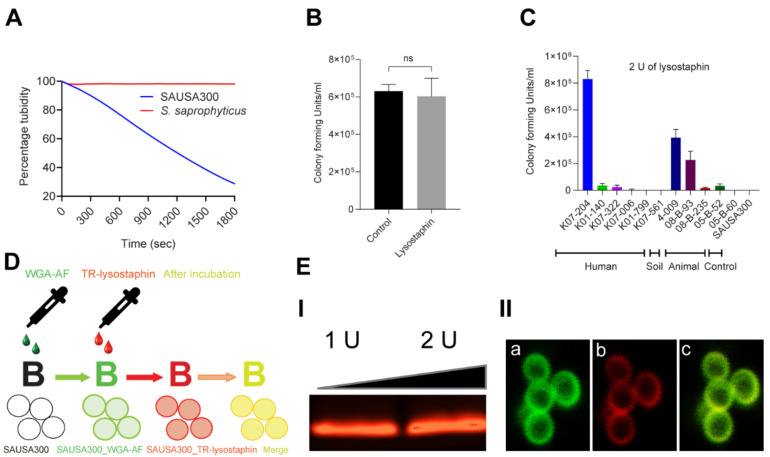
Lysostaphin resistance pattern in ST72 isolates and the efficiency of lysostaphin binding to the cell wall of *Staphylococcus aureus.* (**A**) Lysostaphin mediated killing kinetics of lysostaphin susceptible *S. aureus* USA300 (*lys^s^*) in comparison to the lysostaphin-resistant *Staphylococcus saprophyticus* (*lys^r^*) using the cell turbidity reduction assay. *S. aureus* USA300 (*lys^s^*) showed 70% reduction of cell turbidity compared to *S. saprophyticus* (*lys^r^*); (**B**) The lysostaphin resistance of *S. saprophyticus* (*lys^r^*) was further confirmed by colony forming unit (CFU) counting without (control) and with lysostaphin treatment, showing no significant difference in CFU counts; (**C**) Differential resistance pattern in 11 isolates of *S. aureus* ST72 against 2 U of lysostaphin upon 5 min of incubation wherein K07-204 (human), 4-009 (soil), and 08-B-93 (animal) showed lysostaphin resistance compared to lysostaphin-susceptible *S. aureus* USA300 (control); (**D**) Schematic diagram displays lysostaphin binding to the cell wall labeled with wheat germ agglutinin Alexa Fluor 488 (WGA-AF) (green fluorescence) with colocalized Texas Red labeled lysostaphin (red fluorescence of TR-lysostaphin); and (**E**) (**I**) Texas Red-labeled lysostaphin on agarose gel showing the red fluorescent protein band, (**II**) Colocalization of TR-lysostaphin on WGA-AF labeled green fluorescent cell wall of lysostaphin resistant human isolate of ST72 K07-204 wherein (**a**) the green channel of confocal photomicrograph shows WGA-AF labeled green fluorescent cell wall boundary of staphylococcal cell, (**b**) the red channel of the confocal photomicrograph shows the red fluorescent cell wall upon TR-lysostaphin binding, and (**c**) the merged channel of green (**a**) and red (**b**) shows the yellow fluorescent cell boundary, confirming the efficient binding of lysostaphin with the staphylococcal cell wall.

*S. aureus* possesses a thick peptidoglycan responsible for the maintenance of cell shape and integrity [30]. Lysostaphin is known to specifically target and lyse *S. aureus* cells by cleaving the pentaglycine bridges in the peptidoglycan layer of the cell wall [31], and is thus considered to be a potent enzybiotic. As growing evidence supports the hypothesis that ST72 isolates are resistant to various antibiotics, it is necessary to test whether lysostaphin can be used for treating AMR ST72 infections. Therefore, we evaluated the susceptibility of ST72 isolates (Table S1) to lysostaphin against that of lysostaphin-susceptible *S. aureus* USA300 (hereinafter referred to as SAUSA300) [32] and lysostaphin-resistant *Staphylococcus saprophyticus* [33] (Figure 1A,B). The time-dependent turbidity reduction assay was used to test lysostaphin susceptibility over a 30 min period. The loss of turbidity represents the complete loss of cellular integrity due to the collapse of cell wall architecture of SAUSA300 (Figure 1A) compared to *S. saprophyticus* (Figure 1B). Treatment with 2 U of lysostaphin eradicated most of the *S. aureus* ST72 isolates and SAUSA300. However, the ST72 isolates from human K07-204, animal 08-B-93, and soil 4-009 showed differential resistance to lysostaphin (Figure 1C). The percent loss of turbidity was up to 37% in the case of K07-204 (lysostaphin resistant, *lys^r^*) compared to 60% in K07-561 and SAUSA300 (lysostaphin-susceptible, *lys^s^*) strains, wherein the initial turbidity (OD_600_ = 1) was considered as 100% (Figure S1). Out of 11, K07-204 (human isolates) and 4-009 (soil) were chosen for further experiments to ensure their lysostaphin resistance wherein the lysostaphin-susceptible (SAUSA300) and -resistant (*S. saprophyticus*) strains were used as controls.

K07-204, the human isolate with the highest degree of lysostaphin resistance, can provide answers to two pertinent questions, i.e., (a) do the ST72 resistant/susceptible isolates possess similar lysostaphin-binding activity? And (b) does lysostaphin display differential catalytic cleavage activity (CCA) toward the pentaglycine bridges in the cell wall of lysostaphin-resistant versus susceptible ST72 isolates? To validate the interaction of lysostaphin with the cell wall in *lys^r^* K07-204, the colocalization of Texas Red (TR)-labeled lysostaphin (TR-lysostaphin) and wheat germ agglutinin Alexa Fluor 488 (WGA-AF) was analyzed, as depicted in Figure 1D. WGA-AF possesses a lectin residue which is known to bind carbohydrate moieties in the cell walls of Gram-positive bacteria, specifically on N-acetylglucosamine residues. Red fluorescent TR-lysostaphin (Figure 1E(I)) protein colocalized with the WGA-AF labeled cell wall (Figure 1E(IIa)), resulting in yellow fluorescence (Figure 1E(IIc)) compared to the red fluorescence observed upon the binding of TR-labeled lysostaphin (Figure 1E(IIb)). These results clearly indicate that lysostaphin efficiently binds to the cell wall of ST72 K07-204. Therefore, it is expected that lysostaphin would presumably possess differential CCA for the peptidoglycan layer of the *lys^r^* K07-204 human isolate.

### 2.2. Phenotypic Assessment of the Lysostaphin Resistance in ST72 Isolates

**Figure 2:**
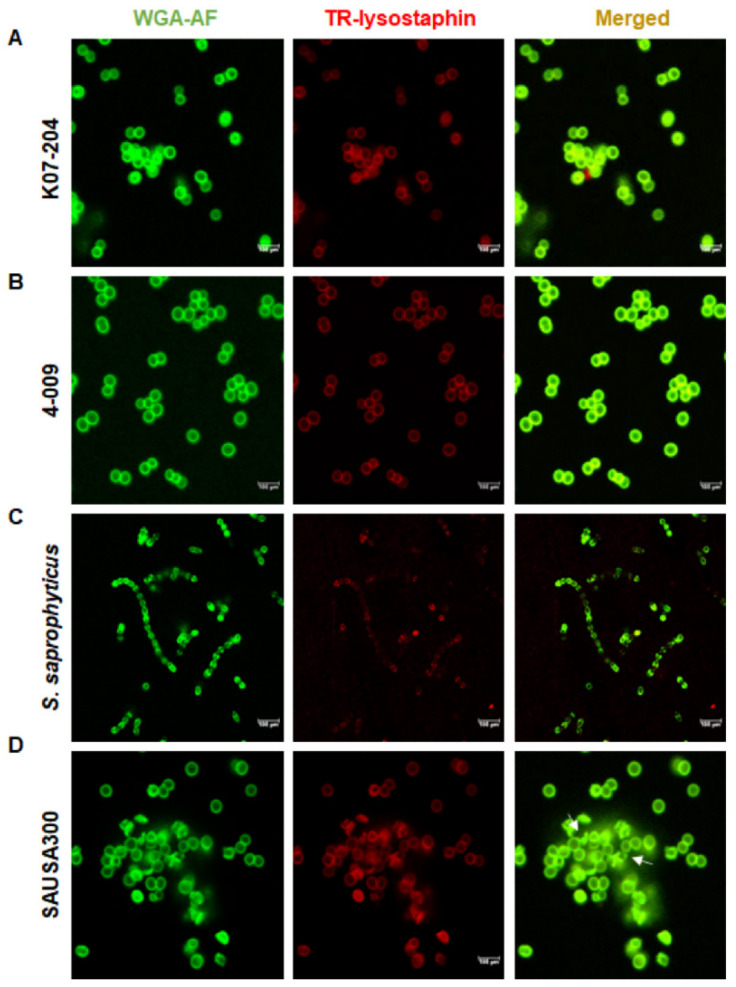
Phenotypic assessment of the lysostaphin-binding and catalytic cleavage activity of lysostaphin resistant isolates of ST72. (**A**–**D**) Confocal microscopic images of lysostaphin-resistant ST72 human isolate K07-204 (**A**); lysostaphin-resistant ST72 soil isolate 4-009 (**B**); lysostaphin-resistant control *S. saprophyticus* (**C**); and lysostaphin-susceptible control *S. aureus* USA300 (**D**) upon treatment with TR-lysostaphin wherein white arrows indicate the broken cells after lysostaphin treatment. TR-lysostaphin binds efficiently with both ST72 isolates and *S. aureus* USA300, while TR-lysostaphin showed the least binding with *S. saprophyticus.*

Confocal laser scanning microscopy (CLSM) was performed to assess the differential catalytic cleavage activity of lysostaphin and the loss of cellular integrity. The staphylococcal cell wall showed a red fluorescence at the bacterial boundary upon TR-lysostaphin-binding without disrupting the WGA-AF labeled green fluorescent cell wall. The colocalization of green and red fluorescence was shown by merged yellowish fluorescent images for K07-204 and 4-009 without visible cell lysis (Figure 2A,B). However, lysostaphin treatment with SAUSA300 resulted in cell lysis, shown by the broken/distorted cells (Figure 2D). These results indicated that lysostaphin can bind to the surface of both the *lys^r^* and *lys^s^* strains of *S. aureus*, but the catalytic cleavage response was found to be lower in the case of *lys^r^* strains, resulting in differential cell lysis. Surprisingly, TR-lysostaphin was found to interact weakly with the cell wall of *S. saprophyticus* (Figure 2C), indicating architectural difference in the peptidoglycan structure of *S. saprophyticus* compared to that of *S. aureus* strains. Thus, the resistance of *S. saprophyticus* seems to be primarily due to the lower binding affinity of lysostaphin. 

### 2.3. Confirmation of the Lysostaphin Resistance in ST72 Isolates

**Figure 3:**
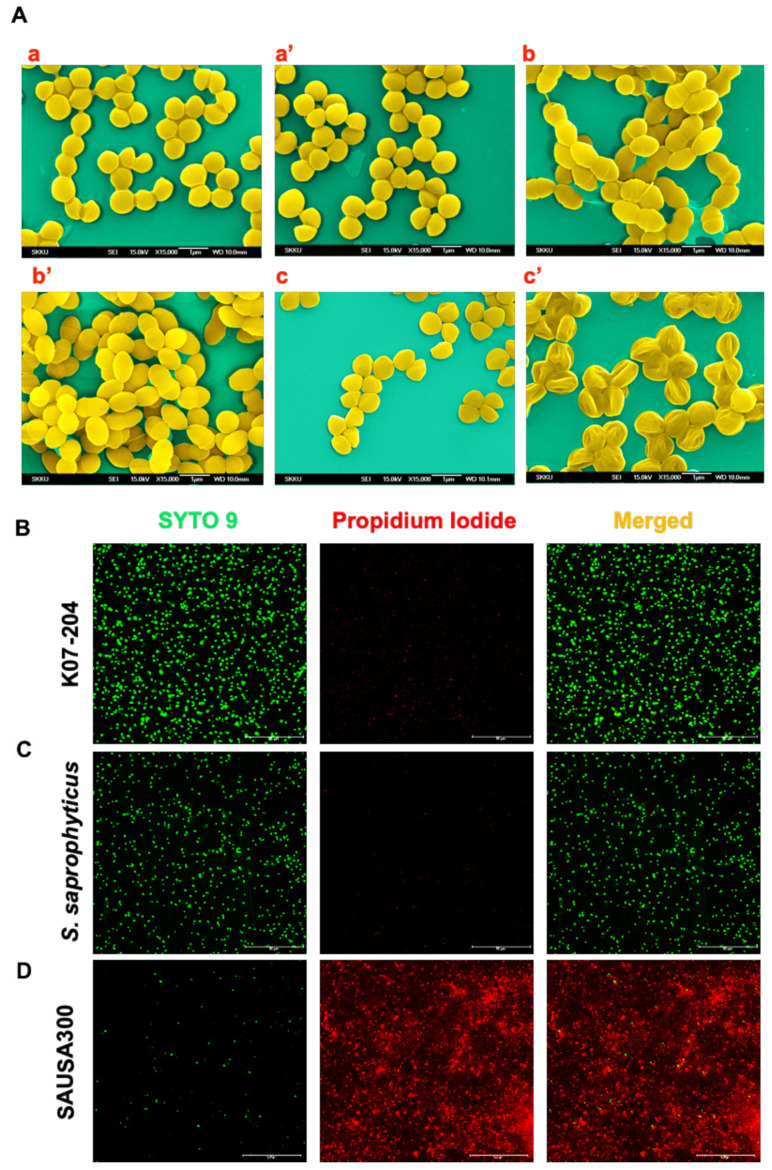
Confirmation of lysostaphin-mediated alteration in the cell wall and consequent cell death. (**A**) Scanning electron microscopy (SEM) to visualize the alteration in the cellular morphology showing staphylococcal cells without lysostaphin treatment wherein K07-204 (**a**); *S. saprophyticus* (**b**); and SAUSA300 (**c**); and after lysostaphin treatment K07-204 (**a’**); *S. saprophyticus* (**b’**); and SAUSA300 (**c’**). Both K07-204 and *S. saprophyticus* (*lys^r^*) did not show any alteration, while the SAUSA300 (*lys^s^*) cells were shrunk upon lysostaphin treatment due to the catalytic cleavage activity of lysostaphin; (**B**–**D**) Confocal microscopy images of live/dead staining of ST72 resistant isolates and its comparison with SAUSA300 to assess the consequent proportion of live/dead staphylococcal cells, wherein SYTO9 stains the total cells (green), whereas propidium iodide (PI) exclusively stains dead cells (red). Both K07-204 (**B**) and *S. saprophyticus* (**C**) showed a lower number of dead (red) cells compared to SAUSA300 (**D**) upon 2 U of lysostaphin treatment.

Nevertheless, the lysostaphin-induced lysis or alteration in cell wall architecture and resultant changes in cellular phenotype could not be clearly visualized using CLSM imaging primarily due to lower magnification. Therefore, we used scanning electron microscopy (SEM) to observe changes in the cell surface without and with lysostaphin treatment. The SEM images clearly showed that K07-204 and 4-009, *lys^r^* ST72 isolates, possessed intact cell walls in response to lysostaphin treatment (Figure 3A(a’) and Figure S2A’) and were phenotypically comparable to untreated controls (Figure 3A(a) and Figure S2A) and *S. saprophyticus* (Figure 3A(b,b’)). However, the *lys^s^* SAUSA300 cells (Figure 3A(c) shrunk upon lysostaphin treatment resulting in cellular death (Figure 3A(c’)), which was expected due to the known endopeptidase action of lysostaphin [34]. The *S. saprophyticus* cells remained intact upon lysostaphin treatment, as observed by both SEM and CLSM images (Figure 3A(b,b’)). Despite the equivalent interaction of TR-lysostaphin in both ST72 isolates and *lys^s^* SAUSA300, it is evident that the human (K07-204) and soil (4-009) isolates of ST72 showed resistance against lysostaphin due to the differential catalytic cleavage activity of lysostaphin.

To further confirm the alterations in cell wall architecture and the resultant changes in cellular phenotype lead to bacterial cell death, live/dead staining was performed using SYTO9/PI staining, wherein SYTO9 stains the total cells (green), whereas propidium iodide (PI) exclusively stains dead cells (red) (Figure 3B–D). SAUSA300 (*lys^s^*) cells were found to be completely stained with PI (red fluorescent cells), indicating their complete cell death (Figure 3D), while the *lys^r^* isolates of ST72 K07-204, *S. saprophyticus* and 4-009 showed intact cells, confirming lysostaphin resistance in these strains (Figure 3B,C and Figure S2B).

### 2.4. Investigating the Existing Mechanism of Lysostaphin Resistance

*Staphylococcus simulans* biovar *staphylolyticus* is a lysostaphin producing strain, and thus harbors the genes coding for a lysostaphin endopeptidase (*lss)* and a lysostaphin immunity factor (*lif*) on its pACK1 plasmid for protection against its own lysostaphin [18,35] whereas the *lys^s^* SAUSA300 strain lacks these genes. To understand the differential CCA of lysostaphin against ST72, we employed a comparative genomics approach by using lysostaphin to treat lysostaphin-producing, -resistant (*lys^r^*, *S. simulans*), and -susceptible (*lys^s^*, SAUSA300) strains; this enabled us to identify the genes whose presence is directly linked with lysostaphin resistance, e.g., *lss* and *epr* encoding glycylglycine endopeptidase and endopeptidase resistance, respectively. Then, we attempted to identify mutations in genes that are known to contribute to the development of lysostaphin resistance through diverse mechanisms. Next, we evaluated the presence of these genes or mutations in ST72 isolates susceptible or resistant to lysostaphin.

First, the degenerate primers for the amplification and cloning of these genes were designed by collecting the sequences from different staphylococcal genomes (Table S2). *lss* and *epr* genes were absent in ST72 isolates (K07-204 and K07-561), whereas *S. simulans* harbored both *lss* and *epr*, which enabled lysostaphin production and conferred lysostaphin resistance, respectively. These results negated the possibility that ST72 is a lysostaphin producer and harbors an autoimmunity mechanism in *lys^r^* human isolates K07-204 and *lys^s^* K07-561 (Figure S3). The genes that are known to be directly responsible for the development of lysostaphin resistance are *epr*-like genes (*fmhC*/*eprh*) and *femABX* [28,36,37,38]. These isolates harbor *fmhC* in their genome. However, the sequences of the *fmhC* gene responsible for lysostaphin resistance in both *lys^r^* K07-204 and *lys^s^* K07-561 isolates were found to be 100% identical (Figures S4 and S5). 

Second, we targeted *lyrA* and *femABX* that usually play a role in resistance [38,39,40] by altering the cell wall assembly. As mutations in these genes are important for conferring lysostaphin resistance [23,26], full-length *lyrA*, *femA, femB*, and *femX* were amplified, cloned, and sequenced. The sequences obtained were translated and aligned for *lys^r^* K07-204 as well as *lys^s^* K07-561 isolates to identify any mutations responsible for the differential CCA of lysostaphin. The sequencing results clearly demonstrated that their translated amino acid sequences were identical in lysostaphin resistant *lys^r^* K07-204 and susceptible *lys^s^* K07-561 strains (Figures S4 and S5). These results clearly indicated that none of the known mechanisms of lysostaphin resistance exist in *lys^r^* K07-204 (Table 1).

### 2.5. Comparative Genomics Analysis of lys^r^ K07-204 and lys^s^ K07-561

**Figure 4:**
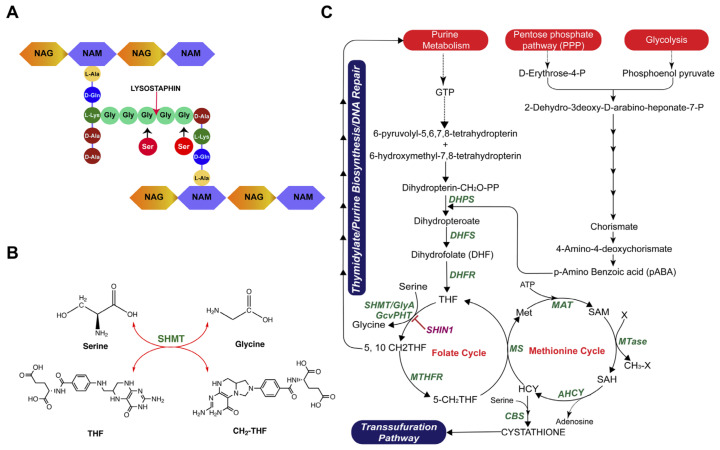
Novel mechanism of lysostaphin resistance in ST72 isolate and its associated metabolic pathway. After screening all the possible genes and mutation(s) known for lysostaphin resistance, no existing mechanisms (genes, mutations) of lysostaphin resistance was found to be functioned in ST72. (**A**) The schematic diagram shows the fundamental reason of lysostaphin resistance in staphylococcal cells due to the modification of glycine residues of pentaglycine bridge to serine; (**B**) Serine hydroxymethyltransferase (SHMT) is an indispensable enzyme for the one-carbon metabolism of serine/glycine interconversion and is linked to folate/methionine cycle. Therefore, *glyA*/*shm*T gene was hypothesized to be involved in lysostaphin resistance. (**C**) The metabolic pathway showing the interdependence of folate/methionine cycle and the key role of *shmT* serine/glycine homeostasis. One-carbon metabolism is responsible for the transfer of methyl group to various substrate and cofactors in folate, methionine cycle, and transsulfuration pathways. Various enzymes are denoted in green font while the substrates are depicted in regular font. The abbreviation used in the pathway wherein enzymes are DHPS (Dihydropteroate synthase); DHFS (Dihydrofolate synthase); DHFR (Dihydrofolate reductase); SHMT (Serine hydroxymethyltransferase); GcvPHT (glycine cleavage system); MTHFR (Methylene tetrahydrofolate reductase); MS (Methionine synthase); MAT (Methionine adenosyltransferase); MTases (Methyl transferases); AHCY (S-adenosylhomocysteine hydrolase), and CBS (Cystathionine beta-synthase) and substrates are THF (Tetrahydrofolate); 5, 10 CH_2_-THF (5, 10 methylene tetrahydrofolate); 5-CH_2_-THF (5-methylene tetrahydrofolate); Met (Methionine); SAM (S-adenosyl methionine); SAH (S-adenosyl homocysteine), and HCY (Homocysteine). One-carbon metabolism is important in cellular homeostasis by maintaining cellular seine/glycine through folate cycle, methionine cycle (protein synthesis), DNA synthesis and repair, redox balance, and various methylation reactions.

After screening all the known genes/mutations associated with various mechanisms of lysostaphin resistance in human isolates of ST72 K07-204 (*lys^r^*) and K07-561 (*lys^s^*) along with control SAUSA300 (*lys^s^*), we had no leads as to how the mechanism of differential lysostaphin response in ST72 works. Therefore, whole-genome sequences of ST72 K07-204 (*lys^r^*) and K07-561 (*lys^s^*) were compared (GenBank Accession no. JACSIU000000000.1 and JACORE000000000.1) for an in-depth comparative and subtractive genomics analysis, which was required to elucidate the unknown mechanism of lysostaphin resistance in *lys^r^* K07-204. Similar levels of binding of TR-labeled lysostaphin to the cell wall in both *lys^r^* K07-204 and *lys^s^* K07-561 indicated the differential CCA of lysostaphin towards the pentaglycine bridge. The differential CCA of lysostaphin towards the pentaglycine bridge is known to occur if the glycine residue is converted to serine [36]. Therefore, we hypothesized that the enzyme involved in serine/glycine conversion might be relevant to the resistance (Figure 4A). To validate this possibility, we constructed a metabolic pathway model of *lys^r^* K07-204 based on comparative genomics analysis. From this metabolic pathway model (Figure 4B), we found that serine hydroxymethyltransferase presumably plays an important role in serine/glycine interconversion with homeostasis of tetrahydrofolate (THF) and 5,10-methylene tetrahydrofolate (MTHF) cellular pool in the folate cycle of one-carbon metabolism (Figure 4B,C). Therefore, we aimed to study the role of the *shmT* gene in glycine/serine interconversion and its possible contributing role in the differential lysostaphin resistance between human isolates, *lys^r^* K07-204 and *lys^s^* K07-561.

### 2.6. Role of shmT in Lysostaphin Resistance

**Figure 5:**
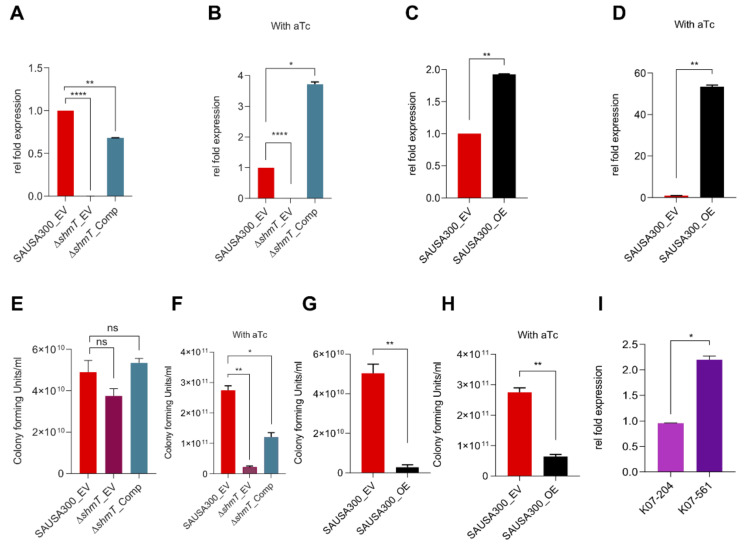
Assessment of *shmT* expression and functional genomics to establish the role of *shmT* in lysostaphin resistance. (**A**–**B**) Gene expression of *shmT* in SAUSA300 recombinant strains including SAUSA300_EV, Δ*shmT*_EV and Δ*shmT*_Comp without induction (**A**), and with anhydrotetracycline (aTc) induction (**B**) wherein no transcript was detected in Δ*shmT* knockout with empty vector (Δ*shmT*_EV) as compared to the wild type *S. aureus* USA300 with empty vector (SAUSA300_EV) and Δ*shmT* complemented strain harboring pRMC2_*shmT* (Δ*shmT*_Comp). The *shmT* expression in (Δ*shmT*_Comp) strain was found to be moderate without aTc induction while it was significantly enhanced (3.5-fold) upon aTc induction. **(C**–**D)** Gene expression of *shmT* in SAUSA300 recombinant strains SAUSA300_EV and SAUSA300_OE constructed by expressing *shmT in trans* (plasmid: *pRMC2_shmT)* under tetracycline inducible promoter in the wild type *S. aureus* USA300. Expression of *shmT* in SAUSA300_OE versus SAUSA300_EV without (**C**) and with aTc induction (**D**) was found to be 2 and 53-fold higher, respectively, than control. (**E**–**F**) Assessment of colony forming unit in SAUSA300 recombinant strains SAUSA300_EV, Δ*shmT*_EV and Δ*shmT*_Comp showing the relative susceptibility of Δ*shmT*_EV strain compared to SAUSA300_EV and Δ*shmT*_Comp strains (**E**) whereas the susceptibility of Δ*shmT*_Comp strain was enhanced upon higher expression of *shmT* using aTc induction (**F**). The susceptibility of Δ*shmT*_EV showed the plausible involvement of *shmT* in lysostaphin resistance. **(G**–**H)** The SAUSA300_OE strain without induction (**G**), and with aTc induction (**H**) showed extreme susceptibility towards lysostaphin as compared to SAUSA300_EV. Both Δ*shmT*_Comp and SAUSA300_OE showed higher susceptibility to lysostaphin as compared to empty vector control SAUSA300_EV upon aTc induction and resultant overexpression of *shmT*; and (**I**) Expression of *shmT* in ST72 isolates K07-204 versus K07-561, wherein the K07-561 showed overexpression of *shmT*, which is the plausible reason of why K07-561 was susceptible to lysostaphin compared to K07-204.

To establish the role of *shmT* in lysostaphin resistance, we investigated the correlation between the *shmT* expression and lysostaphin resistance. For this purpose, we made SAUSA300 with its empty vector (SAUSA300_EV), *shmT* knockout with its empty vector (Δ*shmT_*EV), and Δ*shmT* complemented (Δ*shmT_*Comp.) strains (Table S3). Then, the *shmT* gene expression was quantified by qRT-PCR. While SAUSA300_EV and Δ*shmT* complemented strains showed comparable levels of *shmT* expression without anhydrotetracycline (aTc) induction, no transcript trace was detected in the Δ*shmT* knockout (Δ*shmT_*EV) strain (Figure 5A). The aTc induction enhanced the *shmT* expression in the complemented strain by 3.5-fold compared to the wild type SAUSA300 containing empty vector (SAUSA300_EV) (Figure 5B). Additionally, we made strains with *shmT* overexpression (*shmT*_OE) in the wild type SAUSA300 under aTc inducible promoter for ectopic overexpression of *shmT*. The expression levels of *shmT* without and with aTc induction were found to be 2- and 53 -fold higher, respectively, compared to the empty vector control, SAUSA300_EV (Figure 5C,D).

After assessing the expression level of *shmT* in each strain, its impact on lysostaphin resistance was investigated by the CFU assay using 2 U of lysostaphin treatment. Δ*shmT* _EV was found to be insignificantly susceptible to empty vector control SAUSA300_EV and complement strains (Δ*shmT_*Comp.) without aTc induction (Figure 5E). Interestingly, the enhancement in *shmT* expression with aTc induction showed a significant susceptibility of Δ*shmT* _EV compared to empty vector control SAUSA300_EV and complement strains (Δ*shmT_*Comp.) (Figure 5F). The CFU assay was also conducted using *shmT*_OE both without and with aTc induction (Figure 5G,H). Paradoxically, the ectopic overexpressing of *shmT* in the wild type strain showed a higher level of susceptibility toward lysostaphin compared to SAUSA300_EV (Figure 5G,H). These results showed that overexpression of the *shmT* gene induces a higher level of susceptibility towards lysostaphin as compared to the uninduced control. To assess the correlation between *shmT* gene expression and lysostaphin resistance/susceptibility between the *lys^r^* and *lys^s^* human isolates of ST72, native *shmT* expression was monitored. A 2-fold higher expression of the *shmT* gene was observed in *lys^s^* isolate K07-561 than in *lys^r^* K07-204 isolate (Figure 5I)_._ These results suggest that the higher level of *shmT* expression is the reason for the lysostaphin susceptibility of K07-561 compared to K07-204, which is in complete agreement with the results of *shmT*-overexpressing strain of SAUSA300.

Serine hydroxymethyltransferase (SHMT) (EC 2.1.2.1) is a ubiquitous and extensively studied pyridoxal 5′-phosphate- (PLP dependent) enzyme in all domains of life from bacteria to humans [41]. Two SHMTs, SHMT1 (cytosolic SHMT, GlyC) and SHMT2 (Mitochondrial SHMT, GlyM), are known to be present in humans. The human cytosolic and mitochondrial SHMT*s* displayed 45.5% and 42.0% identity with SHMT of *S. aureus* USA300, respectively (Figure S6). A small molecule denoted as SHIN1 (serine hydroxymethyltransferase inhibitor 1) is known to target human SHMT [42]. Therefore, it is expected that the human SHMT inhibitor, SHIN1, would be more likely to work as an inhibitor against staphylococcal SHMT. Interestingly, the phenotypic assessment of lysostaphin resistance/susceptibility of K07-204 upon SHIN1-mediated inhibition of SHMT showed the slightly enhanced resistance of K07-204 to lysostaphin while the overexpression of *shmT* reduced the lysostaphin resistance of K07-204 (Figure S7). Collectively, these results confirmed the contributing role of *shmT* in lysostaphin resistance/susceptibility both in SAUSA300 as well as in the *lys^r^* K07-204 ST72 isolate (Figure 5).

### 2.7. Role of shmT in Maintenance of Virulence Potential of S. aureus

**Figure 6:**
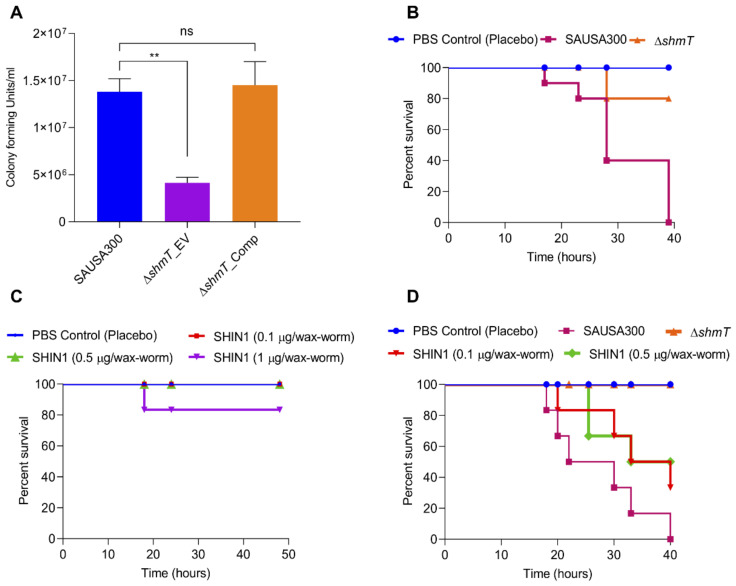
Validating the role of *shmT* in virulence potential of SAUSA300 using in vitro mammalian cells, and in vivo wax-worm infection model. (**A**) Assessment of internalization (invasion/phagocytosis) and survival potential of SAUSA300, Δ*shmT* knockout and Δ*shmT* complemented strains under in vitro mammalian cell culture conditions using murine macrophage, RAW264.7 cells. The Δ*shmT* knockout showed significantly reduced survival inside the macrophage as compared to wild type SAUSA300 and Δ*shmT* complemented strains; (**B**) Survival graph for wax-worms infected by SAUSA300 and Δ*shmT* knockout (2.0 × 10^5^ bacterial cells). The number of wax-worms in each group was 10 (*n* =10). (**C**) Assessment of toxicity of the SHMT inhibitor (SHIN1) for wax-worms (*n* = 10). Varying concentrations of SHIN1 (0.1 μg, 0.5 μg and 1μg in 20 μL solution) were injected into the wax-worms and the survival of the worms was observed for up to 80 h along with 20 μL placebo PBS control. The SHIN1 did not show any toxicity to the worms up to 0.5 μg; and (**D**) The treatment of SHIN1 inhibitor protected 50% wax-worm infected by the wild type SAUSA300, indicating that SHIN1 inhibits the pathogenesis of the wild type SAUSA300.

In general, the *shmT* gene has the role of tetrahydrofolate cycle in one-carbon metabolism [43,44] which is a key pathway, important for folate metabolism, DNA synthesis and repair, methionine biosynthesis, and maintenance of redox status of the cells (Figure 4C). Therefore, we compared the internalization (invasion and phagocytosis) potential of recombinant strains to uncover any possible role of the *shmT* gene in the virulence potential of SAUSA300. Interestingly, the Δ*shmT* knockout showed a decrease in intracellular bacterial cells as compared to the wild type SAUSA300*,* indicating that the *shmT* gene could contribute to the survival of SAUSA300 inside the host cells, and therefore, possibly plays an important role in that the *shmT* gene could contribute to the survival of SAUSA300 inside the host cells, and therefore possibly plays an important role in the virulence and pathogenesis of *S. aureus* (Figure 6A).

Furthermore, to validate the in vitro infection results, knockout strains were injected into wax-worms (*n* = 10) to compare the role of *shmT* in the role of shmT in the pathogenic potential of SAUSA300. The in vivo infection experiment showed ≥80% survival of Δ*shmT* knockout in several experiments*,* whereas the wild-type SAUSA300 resulted in 100% mortality of wax-worms within 40 h of infection (Figure 6B). These results indicate that *shmT* is one of the most potent virulence factors and can be used as a novel drug target for hypervirulence SAUSA300 against the host. The role of *shmT* on the fitness of SAUSA300 was assessed by comparing the growth of wild type SAUSA300 and Δ*shmT* knockout in TSB media for 16 h. The growth of the Δ*shmT* knockout and wild type SAUSA300 was found to be comparable (Figure S8), indicating that *shmT* does not play any major role in the bio-fitness of *S. aureus* strains under free-living conditions. To test the feasibility of SHIN1 functionality for protecting the wax-worm during in vivo infection conditions, we tested the toxicity of the SHIN1 at varying concentrations with both the bacteria (0.1, 0.2, 0.5, and 1, and 5, 7, and 10 μg/mL) and wax-worm (0.1, 0.2, 0.5, and 1 μg/wax-worm). The SHIN1 showed insignificant inhibition of bacterial growth up to 2 μg/mL (Figure S9) and were found to be non-toxic up to 0.5 μg/ wax-worm (Figure 6C). The in vivo infection of wild type SAUSA300 to wax-worm could kill 100% of worms within 40h of infection while the SHIN1 (0.5 μg/wax-worm) showed ≥50% protection of wax-worms infected with the same number of wild type SAUSA300 as compared to the Δ*shmT* knockout and PBS (placebo) control group (Figure 6D). Both the in vitro and in vivo infection results clearly demonstrated that the *shmT* is a potent antivirulence drug-target.

## 3. Discussion

The multiple drug resistant *S. aureus* bacterial pathogen [45,46] has not only created mild to lethal infection at the community level but has become an inevitable source of nosocomial infections worldwide [47]. The drug resistance and virulence of MRSA are continuously increasing, primarily due to the continuous acquisition of new antibiotic resistance and virulence genes, which facilitate the clonal selection of AMR strains in hospital settings and highly detrimental virulence determinants for efficient invasion/evasion and/or tolerance/adaptation inside the host cells. The acquisition of antibiotic resistance genes is due to mobile genetic element transposons and/or insertion elements through horizontal gene transfer [48]. Point mutations or the accumulation of single nucleotide polymorphism (SNP) have further enhanced the resistance of pathogens to antibiotic resistance by altering drug targets [49,50]. Despite the ongoing advancements in the discovery of novel antibacterial drugs, antibiotic-based eradication strategies for AMR pathogens are becoming inefficient in complete clearance of infection, resulting in intracellular bacterial communities causing secondary infections [51] due to the development of tolerance or resistance by modulation of gene expression and metabolism.

To solve the bacterial resistance problem, the enzybiotic approach has gained prominence as an alternative because of its efficacy and specificity against MDR bacteria [52]. Among enzybiotics, lysostaphin is successfully (phase II clinical trial) being used against *S. aureus* for both therapeutic and preventive purposes [23,53,54]. Lysostaphin is a 27 kDa secretory enzyme that breaks interlinking pentaglycine peptide bridges of peptidoglycan [19], resulting in the loss of cell wall integrity and consequent bacterial cell death. However, lysostaphin resistance has developed in *S. aureus* strains, including the human isolate of ST72 K07-204 reported in the current study. The lysostaphin resistance known so far is caused by multiple events, possibly as the evolutionary bypass tactic(s) of *S. aureus* against lysostaphin, such as (a) null *femAB* mutants making monoglycine bridges [55], (b) transposon insertion (*lyrA*) [40], (c) acquisition of lysostaphin resistance genes, *epr* [56] and/or (d) *lif* resulted in a 2 to 35% increase in the serine/glycine ratio in peptide bridges of *S. aureus* [37]. However, these known resistance mechanisms were found to be absent in ST72 K07-204. Therefore, K07-204 seems to harbor a novel resistance mechanism that must be investigated.

In the current study, we identified and established that *shmT* can act as a candidate gene to trigger lysostaphin susceptibility against lysostaphin-resistant *S. aureus*. We observed that the enhanced expression of the *shmT* gene translates into a higher susceptibility to lysostaphin. The knockout of *shmT* resulted in enhanced lysostaphin susceptibility in the model SAUSA300. In the Δ*shmT_EV* (knockout with empty vector), the relative reduction in CFU counts showed that resistance to lysostaphin demands a basal level expression of *shmT* (Figure 5A,E). Surprisingly, the overexpression of *shmT* under tetracycline inducible promoter (aTc, anhydrotetracycline) (Figure 5B,D) made SAUSA300 extremely susceptible to lysostaphin (Figure 5F,H), which indicates that optimal expression of the *shmT* gene is required to maintain the homeostasis of metabolites that translate into lysostaphin resistance/susceptibility. Consistently, it was confirmed that K07-204 (*lys^r^*), which is resistant against lysostaphin, showed two-fold higher expression of s*hmT* than lysostaphin susceptible K07-561 (*lys^s^*) (Figure 4I).

As *shmT* is a key gene controlling the one-carbon metabolism pathway, which synthesizes purines, methionine, thymidylate, and glycine in bacterial cells, we aimed to assess the role of *shmT* and its folate metabolism pathway in conjunction with the pathogenesis of WT SAUSA300. The results obtained under in vitro experiments showed that the Δ*shmT* knockout strain has a reduced infection potential compared to WT SAUSA300 and complemented strain of Δ*shmT_*Comp. When wax-worms were injected with equal numbers of cells of WT SAUSA300 or Δ*shmT* stains, the Δ*shmT* knockout showed an extraordinary survival rate of up to 80% compared to SAUSA300, confirming that *shmT* acts as a potent virulence factor; therefore, it can be exploited as an effective drug target to inhibit virulence of *S. aureus* strains. It has also been shown that catfish receiving Δ*shmT* have shown reduced virulence potential as compared to wild type *Edwardsiella ictalurid* [57].

## 4. Materials and Methods

### 4.1. Cells, Chemicals, and Reagents

Mammalian cell lines used in the study were procured from the American Type Culture Collection (ATCC). All the chemicals used in the study were of analytical grade. Lyophilized lysostaphin powder form was purchased (Cat #L7386, Sigma Aldrich, St. Louis, MO, USA) and dissolved in buffer containing 50 mM Tris-HCl and 145 mM NaCl (pH 7.4) as described earlier [20]. The SHMT inhibitor, SHIN1 (Cat #GC32773, GLPBIO, Montclair, CA, USA) was purchased to test its inhibitory effect on staphylococcal SHMT.

### 4.2. Bacterial Cell Growth Conditions

Various wild type staphylococcal strains, including *S. aureus* ST72 isolates (Table S1), were grown on tryptic soy agar (TSA) plates at 37 °C overnight. Bacterial broth culture was grown by inoculating a single colony in tryptic soy broth (TSB) media under orbital shaking (200 rpm) culture conditions at 37 °C for 14–16 h. Bacterial growth was monitored by measuring the optical density at 600 nm (OD_600_) using a spectrophotometer (GE Healthcare, Chicago, IL, USA). Bacterial cells were harvested by centrifugation (3220× *g*) at 4 °C, followed by washing with 1X phosphate buffered saline (PBS, pH 7.2). All the primers used to create recombinant strains are shown in Table S2. The recombinant strains of *S. aureus* USA300 FPR3757 (SAUSA300) (NARSA, USA) harboring either empty vector (pRMC2) or its derivatives were grown in TSA plates and TSB broth media with chloramphenicol at 12.5 µg/mL and 25 µg/mL, respectively. All bacterial strains used in the study are shown in Table S3.

### 4.3. Conjugation of TR-X Succinimidyl Ester with Lysostaphin

Lysostaphin was conjugated with Texas Red (TR, AAT Bioquest, Sunnyvale, CA USA) as described previously [32]. Briefly, 25 U of lysostaphin was subjected to buffer exchange with 100 mM sodium carbonate buffer (pH 10) by centrifugation (17000× *g*) at 4 °C using 3 kDa Amicon ultra centrifugal filters (Merck, Carrigtwohill, Ireland). The buffer exchanged lysostaphin was resuspended in 700 µL (220 nM) of carbonate buffer in which 11.3 µL of TR (3.9 mM stock) was added to maintain a 1:200 lysostaphin to TR ratio. The final reaction volume was maintained at 1 mL. The reaction mixture was continuously mixed overnight at 4 °C using a mixer rocker. Unbound TR was removed by repeated washing with 0.1 M phosphate buffer using 3 kDa Amicon ultra centrifugal filters. Finally, the conjugated TR-lysostaphin was concentrated in 125 µL. Conjugation of lysostaphin and TR was analyzed using SDS-PAGE and was imaged using a gel documentation system (BIO-RAD, USA).

### 4.4. Assessment of ST72 Response to Lysostaphin Treatment

After the cells were harvested, the OD_600_ was maintained at 0.01 in 1× PBS (1 × 10^7^ cells). Cells were treated with 2 U of lysostaphin at 37 °C for 5 min. After treatment, the lysostaphin activity was immediately increased by treatment with 10 µM of phenanthroline which chelates the zinc ion. Both the untreated control and treated cells were serially diluted, and various dilutions were plated on TSA plates to count the CFU.

### 4.5. Turbidity Reduction Assay

To estimate the killing kinetics of lysostaphin, K07-204, K07-561, SAUSA300, and *S. saprophyticus* were grown for 6 h from the overnight grown cultures. PBS-washed bacterial cells were treated with 5 U of lysostaphin and the killing-kinetics were measured for 30 min using a spectrophotometer (V730 JASCO, Tokyo, Japan). After lysostaphin treatment, the killing rate was measured in terms of turbidity reduction for all staphylococcal strains.

### 4.6. Evaluation of Lysostaphin Binding to the Cell Wall of ST72 Isolates

All bacterial strains were grown in TSB to the logarithmic phase. The harvested bacterial cells were washed with 1× PBS, and the OD_600_ was maintained at 0.5 (~5.0 × 10^8^ cells/mL). Cells were labeled with wheat germ agglutinin Alexa Flour stain (WGA-AF) according to the manufacturer’s instructions (ThermoFisher Scientific, Carlsbad, USA), and unbound dye was washed using 1× PBS. The WGA-AF-labeled bacterial cells were incubated with TR-lysostaphin for a short time (5 s) and phenanthroline was added immediately. Bacterial slides were prepared by placing 100 µL of the stained culture on grease-free glass slides for confocal imaging (LSM 510 Meta, Carl Zeiss, Oberkochen, Germany).

### 4.7. Assessment of Lysostaphin Endopeptidase Activity on ST72 Isolates Using SEM

Bacterial strains were treated with 2 U of lysostaphin for 5 min at 37 °C. Cells were fixed in 2% glutaraldehyde (Glutaraldehyde solution Grade I, 8% in H_2_O, Sigma) overnight at 4 °C. After overnight fixation, the cells were washed with PBS twice and dehydrated in an ethanol gradient of 10%, 20%, 40%, 60%, 80%, and finally with 100% ethanol. Cells were mounted on silicon wafers and dried before subjecting to SEM. After drying, platinum sputtering was performed to visualize samples by SEM (FESEMII/EDS/EBSD, JSM700, Jeol, Peadbody, MA, USA).

### 4.8. Live/Dead Staining

ST72 isolates K07-204, K07-016, *S. simulans*, and SAUSA300 were grown until the log phase, and the turbidity of the cells was maintained at OD_600_ equivalent to one. Cells were treated with 5 U of lysostaphin for 5 min at 37 °C. Cells were washed and stained with Live/Dead Baclight Bacterial Viability Kit (Cat #L7007; Invitrogen, Carlsbad, CA, USA). SYTO9 stained all the cells green, whereas PI exclusively stained the dead cells red. After staining, the cells were washed twice with PBS and imaged using confocal microscopy to measure lysostaphin endopeptidase activity-mediated cell death.

### 4.9. Evaluation of Presence/Absence and Mutational Analysis of Known Genes Responsible for Lysostaphin Resistance

To analyze the presence or absence of lysostaphin resistance genes, we designed degenerate primers because the genome sequences were not available during the unfolding of the mechanism of lysostaphin-resistance (K07-204, *lys^r^*) and susceptible K07-561 (lysostaphin-susceptible, *lys^s^*) of ST72. Sequence information of all genes was collected from Uniprot. Sequence alignment was achieved using the multiple sequence alignment tool CLUSTALW. Degenerate primers were designed after finding regions with maximum conserved sequences. The sequences of degenerate primers are shown in Table S2. The *epr*, *lss*, and *fmhC* genes in ST72 isolates were PCR-amplified using degenerate primers to explore the presence or absence of genes related to the known mechanisms of lysostaphin resistance. To identify mutations in genes that are connected to the lysostaphin resistance, the *femAB* family members *femA*, *femB*, *femX*, and *lyrA* [39,40] were PCR-amplified using CN1 genome [58] based primers (Table S2). In this comparative analysis, SAUSA300 was used as a control and K07-204 and K07-561 as lysostaphin-resistant and susceptible isolates, respectively. The *femA*, *femB*, *femX*, and *lyrA* genes were PCR amplified and cloned into TOPO-TA vector (TOPO^TM^ TA Cloning^TM^ kit, Cat #450641, Invitrogen). The recombinant pCR2.1 TOPO vectors harboring the insert fragment were transformed into *E. coli* DH5α and putative clones were picked by blue-white selection on an LB agar plate containing X-gal (25 μg/mL), IPTG (1 mM), and kanamycin (50 μg/mL) plates. The plasmids from the positive clones were isolated using a plasmid purification kit (Qiagen, Hilden, Germany). Both strands were sequenced to achieve the complete sequence of *femA*, *femB*, *femX*, and *lyrA* to analyze the mutation(s), if any. After obtaining nucleotide sequences of the genes, their sequence was translated using the Expasy translation tool. Amino acid sequences of the respective genes were aligned using multiple alignment tools to identify any mutations in the amino acid sequence, which could lead to lysostaphin resistance.

### 4.10. Comparative Genomics-Based Metabolic Pathway Modeling

To further identity the lysostaphin-resistance responsible genes, the genomes of K07-204 (*lys^r^*) and K07-561 (*lys^s^*) (JACSIU000000000.1 and JACORE000000000.1, respectively) [59] were compared with those of the model organism *S. aureus* USA300. The homology model was constructed based on comparative genomics to generate a hypothesis about the possible involvement of one carbon metabolism in serine/glycine homeostasis and its plausible involvement in lysostaphin resistance. The role of the folate cycle in relation to *shmT* in antifolate antibiotic resistance and virulence was studied based on the genomics model.

### 4.11. Functional Genomics of the shmT Gene from K07-204 and Cloning into pRMC2 Vector

To prove the hypothesis that the *shmT* gene might contribute to the biosynthesis of amino acids, and specifically to the interconversion of serine to glycine in the tetrahydrofolate pathway [60], a functional genomics approach was employed. The *shmT* gene of K07-204 was PCR-amplified and cloned into the pCR2.1TOPO cloning vector and sequenced. The resultant pCR2.1TOPO_*shmT* vector was restriction digested using *Kpn1* and *EcoR1* (New England Biolab, MA, USA). The *KpnI-EcoR1* digested *shmT* gene gel was purified and sub-cloned at the same sites in the *E. coli*–*S. aureus* shuttle vector, pRMC2. The empty vector pRMC2 and *shmT* recombinant plasmids pRMC2_*shmT* were transformed into *E. coli* DH5α and selected on LB agar plates supplemented with chloramphenicol (25 µg/mL). The plasmids pRMC2 and pRMC2_*shmT* were isolated and electroporated into *S. aureus* RN4220, followed by WT *S. aureus* USA300 and Δ*shmT* knockout.

The electrocompetent *S. aureus* strains were prepared by washing with cold sucrose solution. Briefly, the electrocompetent cells of *S. aureus* strains (*S. aureus* RN4220, WT *S. aureus* USA300*, and* Δ*shmT* knockout) were prepared using 20 mL log-phase grown cells (OD_600_ 0.6–0.8) in TSB. After centrifugation (3220× *g*) at 4 °C, cell pellets were washed twice with 20 mL of 200 mM sucrose solution, and finally resuspended in 2 mL GTY media (glucose 1 g/L, tryptone 5 g/L, yeast extract 2.5 g/L, pH 7.2) for electroporation. First, the empty vectors pRMC2 and pRMC2_*shmT* were electroporated into *S. aureus* RN4220 electrocompetent cells. Electroporation for all *S. aureus* strains was performed in an electric field of 22 kV/cm, 25 µF capacitance, and 200 Ω resistance using a 1 mm gap electroporation cuvette [61] (Gene Pulser, Bio-Rad, Hercules, CA, USA). The putative recombinants of all *S. aureus* strains were selected on a TSB plate supplemented with chloramphenicol (25 µg/mL). The plasmids (pRMC2 and pRMC2_*shmT*) were extracted from *S. aureus* RN4220 and were finally electroporated in the WT *S. aureus* USA300 and Δ*shmT* knockout. The list of recombinant strains is shown in Table S3.

### 4.12. Quantitative RT-PCR for Expression Analysis of shmT

Total mRNA was isolated from recombinant strains of SAUSA300 and ST72 isolates using the Qiagen RNeasy Mini Kit according to the manufacturer’s protocol. The total mRNA was treated with DNase I to remove any DNA contamination. The DNase I-treated mRNA samples were subjected to PCR amplification of the target gene to confirm the absence of any traces of genomic DNA contamination. Random hexamers premix (RNA to cDNA EcoDry^TM^ premix, Takara Bio, Kusatsu, Japan) was used to prepare cDNA from the isolated mRNA. The primers for qRT-PCR were used with annealing temperatures of 55 ± 2°C and an amplified product size of 200 bp (Table S2). qRT-PCR was performed using SYBR Green Supermix (Bio-Rad), wherein the *gyrA* gene was kept as a housekeeping gene. Expression of the *shmT* genes was normalized with that of the endogenous *gyrA* gene. Relative gene expression was analyzed using the 2^−ΔΔCT^ method [62]. Three independent qRT-PCR experiments were performed, and the statistical significance was calculated by Student’s t-test (*p* < 0.05).

### 4.13. Evaluation of Virulence in ST72 Isolates

The virulence and pathogenic potential of *S. aureus* strains were assessed both under in vitro mammalian culture conditions and using an in vivo waxworm (*Galleria mellonella*) infection model.

- In vitro infection experiment. To assess the role of the *shmT* gene in the virulence of *S. aureus*, an in vitro infection experiment was performed using HEK293 and RAW264.7 mouse macrophage cell lines. Mammalian cells were grown in Dulbecco’s modified Eagle’s medium (DMEM) with 10% fetal bovine serum in a 5% carbon dioxide humidified incubator at 37 °C. Cells were split in 6-well plates (1.0 × 10^6^ cells/well) and incubated for 24 h. After washing with 1× PBS, bacterial cells were provided with invasion medium (DMEM without FBS) before 2 h of infection. The RAW264.7 and HEK293 cells were infected with SAUSA300 strains at *moi* 10 (multiplicity of infection 10) for 30 min as described previously [32]. Extracellular cells were killed using lysostaphin (5 U) and gentamicin (400 µg/mL). Cells were washed three times with 1× PBS to remove residual antibacterial agents. Cells were collected by trypsinization and collected by centrifugation. The cell pellet was washed once again with 1× PBS and treated with 0.04% Triton-X100 to break open the mammalian cells to recover the intracellular bacterial cells. Intracellular bacterial cells were diluted in 1× PBS and the bacterial count was assessed by dilution plating of 100 µl on TSA plates for enumeration of CFU.
- *Galleria mellonella* infection model. Wild-type SAUSA300 and its isogenic *shmT* knockout strains were grown overnight in TSB media under orbital shaking (200 rpm) culture conditions at 37 °C. The overnight grown culture was reinoculated at 100-fold dilution in TSB for 6 h. Bacterial cells were collected by centrifugation (3220× *g*, 4 °C) and washed once with 1 × PBS. The cell number was maintained by adjusting the optical density (OD_600_ = 1) in 1 × PBS. *G. mellonella* were utilized within a week of their receipt. Each group of control or treatment contained 10 wax-worms. Wax-worms were injected with 2.0 ×10^5^ cells (20 µL) in the last left posterior leg using a 0.3 mL syringe (Becton, Dickinson and Company, Franklin Lakes, NJ, USA) and incubated at 37 °C for observation. In each experiment, a group of worms was kept as control and injected with 20 µl 1 × PBS. Worms were observed for survival at different time intervals and the experiment was terminated within 20 h.

## 5. Conclusions

The outcome of this study not only revealed the novel mechanism of lysostaphin resistance, but also conclusively proved that SHMT can act as a potent antivirulence target. We identified and established the role of *shmT* in the lysostaphin resistance of K07-204. Next, we confirmed that SHMT acts as a potent virulence factor in *S. aureus* USA300. Finally, the in vivo experiment with the SHMT inhibitor, SHIN1 showed effective inhibition of the pathogenesis of *S. aureus* USA300. Therefore, designing an array of specific and potent inhibitors specific to bacterial SHMT will not only make *S. aureus* susceptible to antifolate antibiotic drugs and increase their efficacy, but also act as a potent antivirulence drug that would help the host immune system to easily eradicate MDR-MRSA infections.

## Figures and Tables

**Table 1:** Screening of existing mechanisms of lysostaphin resistance.

Genes	CN1		K07-204		K07-561		SAUSA300	
	Presence	Mutation	Presence	Mutation	Presence	Mutation	Presence	Mutation
*femA*	√	−	√	−	√	−	√	−
*femB*	√	−	√	−	√	−	√	−
*femX (fmhB)*	√	−	√	−	√	−	√	+2 *
*fmhC*	√	−	√	−	√	−	√	−
*lyrA*	√	−	√	−	√	−	√	−
*epr*	X		X		X		X	
*lss*	X		X		X		X	

* Represents two mutations in *femX* T254A and E261K with reference to ST72 CN1.

## Supplementary Materials

The following are available online at https://www.mdpi.com/1422-0067/21/23/9135/s1.

## Abbreviations

SHMT	Serine hydroxymethyltransferase
SHIN1	Serine hydroxymethyltransferase inhibitor 1
