# Supplementary material for: Functional Identification of Serine Hydroxymethyltransferase as a Key Gene Involved in Lysostaphin Resistance and Virulence Potential of Staphylococcus aureus Strains
Source: Int J Mol Sci. 2020 Nov 30;21(23):9135. doi: 10.3390/ijms21239135 (PMC7731198; doi:10.3390/ijms21239135)
Supplement: Supplementary file 1 [file ijms-21-09135-s001.pdf]

Supporting Information

TITLE

Functional identification of serine hydroxymethyltransferase as a key gene involved in lysostaphin resistance and virulence potential of *Staphylococcus aureus* strains

RUNNING TITLE

Role of *shmT* in lysostaphin resistance and virulence

AUTHORS

Nayab Batool[1], Kwan Soo Ko[2], Akhilesh Kumar Chaurasia[1]* and Kyeong Kyu Kim[1,2]*

[1]Department of Precision Medicine, Institute for Antimicrobial Resistance Research and Therapeutics, Sungkyunkwan University School of Medicine, Suwon 16419, South Korea

[2]Samsung Advanced Institute for Health Sciences and Technology (SAIHST), Samsung Medical Center (SMC), Sungkyunkwan University School of Medicine, Seoul 06351, South Korea

***Corresponding authors**

Akhilesh Kumar Chaurasia (chaurasia@skku.edu)

Kyeong Kyu Kim (kyeongkyu@skku.edu)

PHONE: +82-31-299-6152

FAX: +82-31-299-6159

Content

1. Supplementary figures

Fig S1. Lysostaphin killing kinetics of human isolates of ST72

Fig S2. Scanning electron and confocal microscopy of ST72 resistant soil isolate, 4-009 to assess lysostaphin-mediated alteration in cell morphology and live/dead staining

Fig S3. PCR based screening of presence/absence of *epr* and *lss* genes responsible for lysostaphin resistance in human isolates of ST72

Fig S4. PCR amplification, cloning, sequencing, and screening of associated mutation(s) in other key genes known for lysostaphin resistance

Fig S5. Cloning, sequencing, multiple sequence alignment to assess the mutation(s) upon translated DNA sequences

Fig. S6. Alignment of SHMT from *S. aureus* USA300 with human *SHMTs* to assess the overall similarity and identity

Fig. S7. Role of SHMT in lysostaphin resistance of K07-204 human isolate of ST72

Fig. S8. The role of *shmT* on the fitness of *S. aureus* USA300

Fig. S9. Serine hydroxymethyltransferase inhibitor 1 **(**SHIN1) toxicity to *S. aureus* USA300 cells at varying concentrations

2. Tables

Table S1. wild type *S. aureus* ST72 isolates

Table S2. Primers used in the study

Table S3. Staphylococcal strains/isolates and plasmid used in the study

Supporting Fig. S1

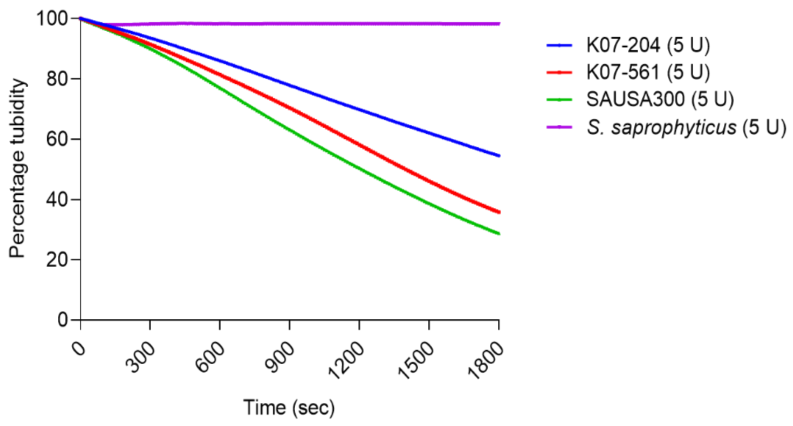

Supporting Fig. S1. Lysostaphin killing kinetics of human isolates of ST72. (A) Lysostaphin mediated killing efficiency using turbidity reduction among *lys^{r}* (K07-204) and *lys^{s}* (K07-561) in comparison to *S. aureus* USA300. Lysostaphin resistant *lys^{r}* K07-204 showed 37% percent turbidity reduction as compared to ≥60% turbidity reduction for K07-561 and *S. aureus* USA300 within 30 min of lysostaphin treatment. *S. saprophyticus* displayed resistance to lysostaphin treatment.

Supporting Fig. S2

A

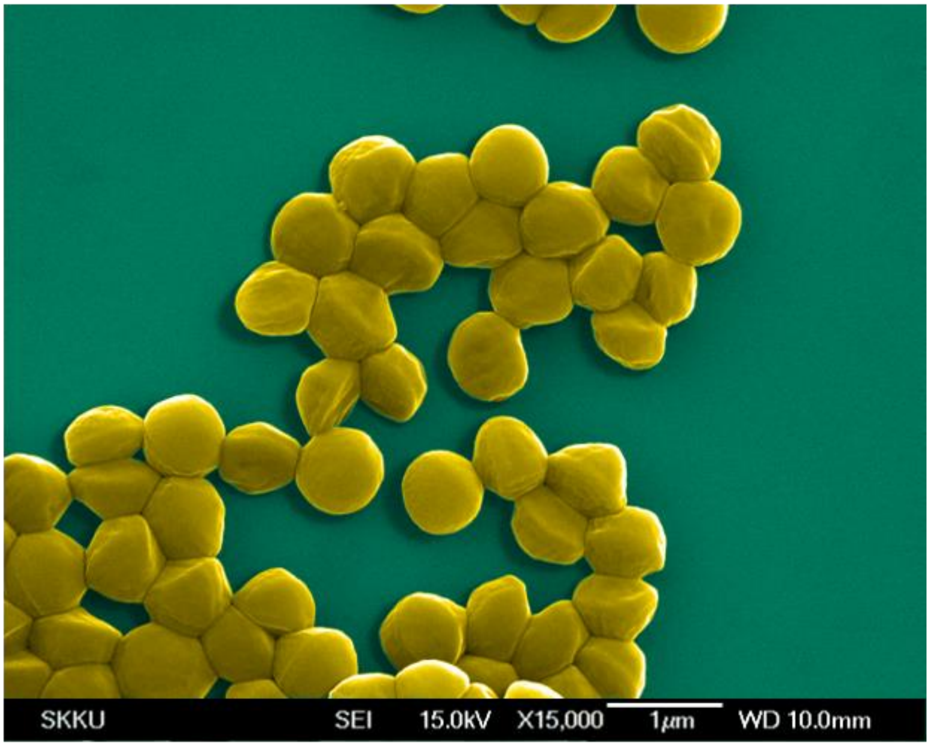

A’

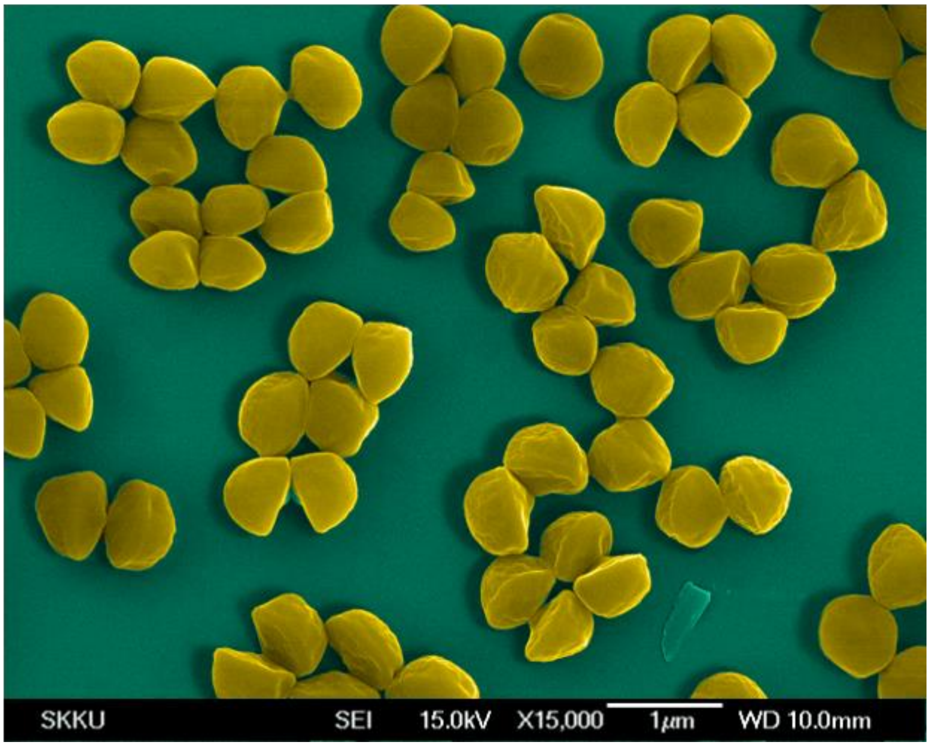

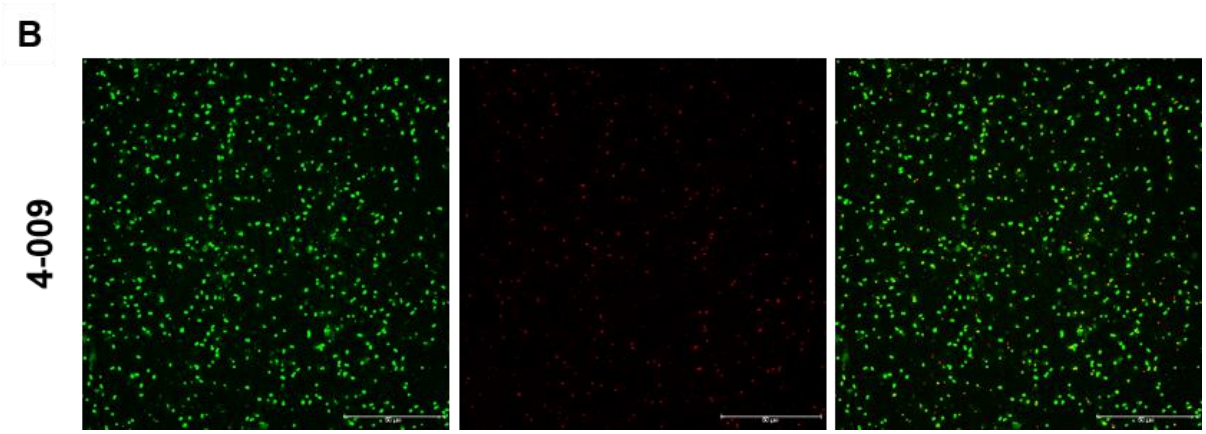

Supporting Fig. S2. Scanning electron and confocal microscopy of ST72 resistant soil isolate, 4-009 to assess lysostaphin-mediated alteration in cell morphology and live/dead staining. **(A-A')** SEM photomicrograph to assess the lysostaphin-mediated alteration in the cell morphology of lysostaphin resistant (*lys*^{r}) ST72 soil isolate 4-009 before (**A**) and after lysostaphin treatment (**A'**) and displayed no alterations post lysostaphin treatment (**A'**); **(B)** Live/dead images of *S. aureus* lysostaphin resistant (*lys*^{r}) ST72 isolate, 4-009 after lysostaphin treatment (4 U) using SYTO9/PI for 5 min. The total number of 4-009 cells were stained with SYTO9 stain (SYTO channel; green fluorescent cells) while a smaller proportion of cells were stained with PI (PI channel; red fluorescent cells) showing dead cells.

Supporting Fig. S3

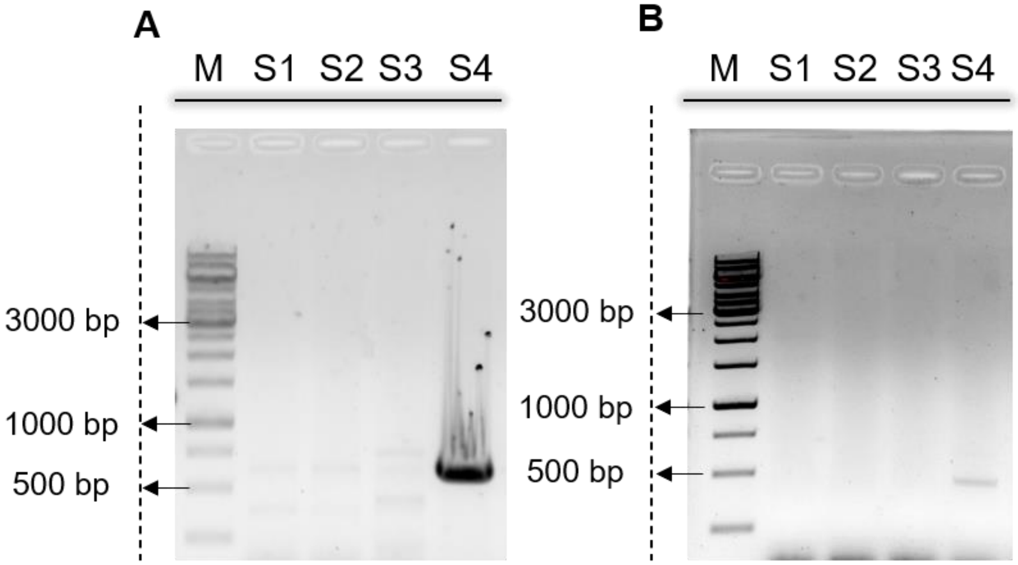

Supporting Fig. S3. PCR based screening of presence/absence of *epr* and *lss* genes responsible for lysostaphin resistance in human isolates of ST72. (A) Agarose gel showing the presence/absence of endopeptidase gene (*epr*) screened by PCR amplification in ST72 isolates, K07-204 (S1), K07-561 (S2) as compared to *S. aureus* USA300 (negative control, S3) and *S. simulans* (positive control, S4) wherein the *epr* gene was amplified only in *S. simulans* on agarose gel conferring lysostaphin protection. **(B)** Agarose gel showing the PCR amplified lysostaphin gene (*lss*) in ST72 isolates, K07-204 (S1), K07-561 (S2) as compared to *S. aureus* USA300 (negative control, S3) and *S. simulans* (positive control, S4) wherein the *lss* gene was amplified only in *S. simulans* conferring lysostaphin production. (M denotes the 1kb DNA marker ranging from 250 bp to 10 kb). These results indicate that the ST72 isolates, K07-204 (*lys*^{r}) and K07-561 (*lys*^{s}) are not the lysostaphin producers.

Supporting Fig. S4

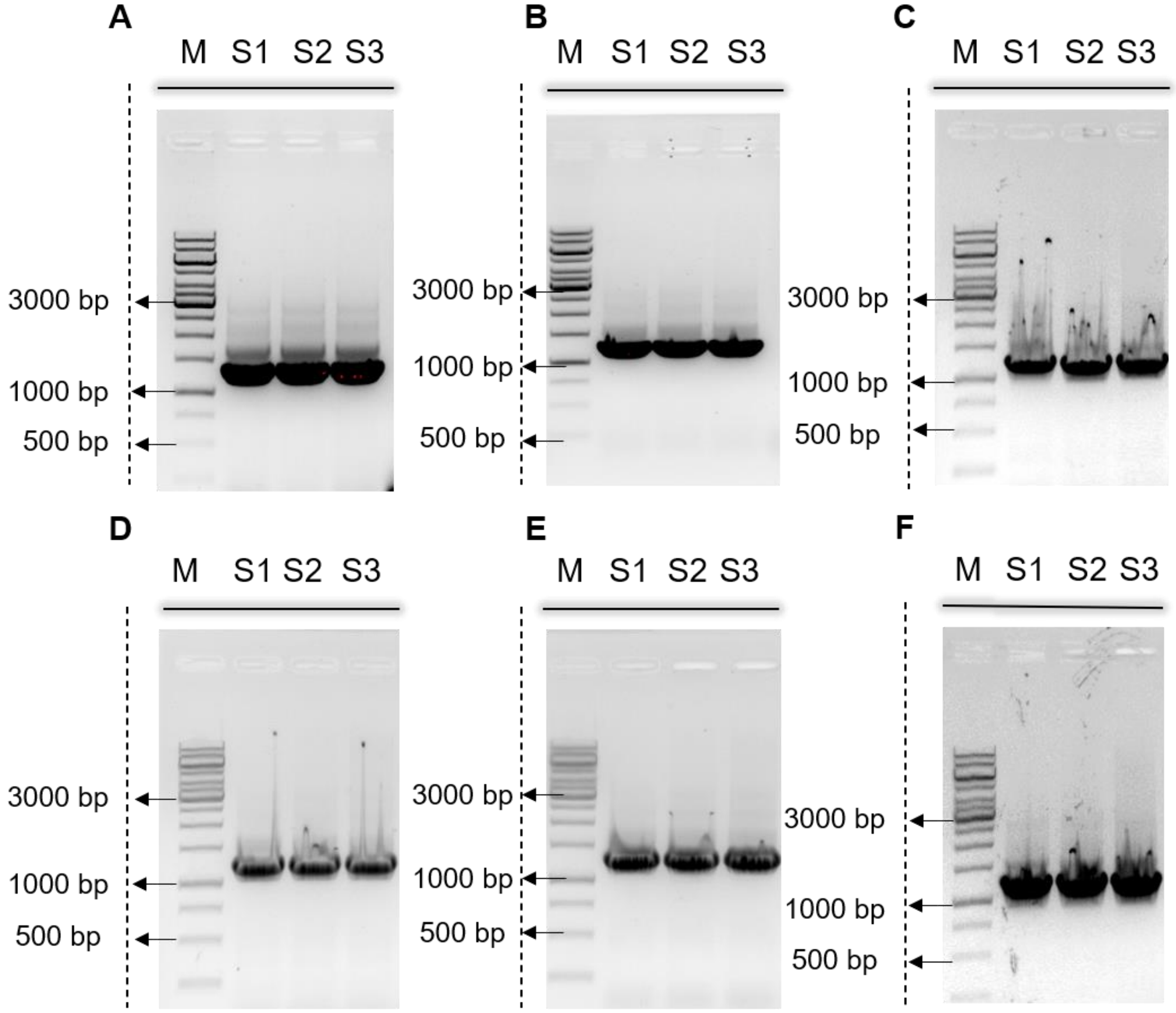

Supporting Fig. S4. PCR amplification, cloning, sequencing, and screening of associated mutation(s) in other key genes known for lysostaphin resistance. (A-F) Agarose gel showing the amplification of *femA* **(A)** *femB* **(B)** *femX* **(C)** *fmhC* **(D)** *lyrA* **(E)** and *shmT* **(F)** in ST72 isolates, K07-204 (S1), K07-561 (S2) and *S. aureus* USA300 (S3). These genes were amplified to clone in pCRTOPO2.1 cloning vector. Clones were sequenced to assess the mutation(s), if any, known to be responsible for lysostaphin resistance. M denotes the 1kb DNA marker ranging from 250 bp to 10 kb.

Supporting Fig. S5

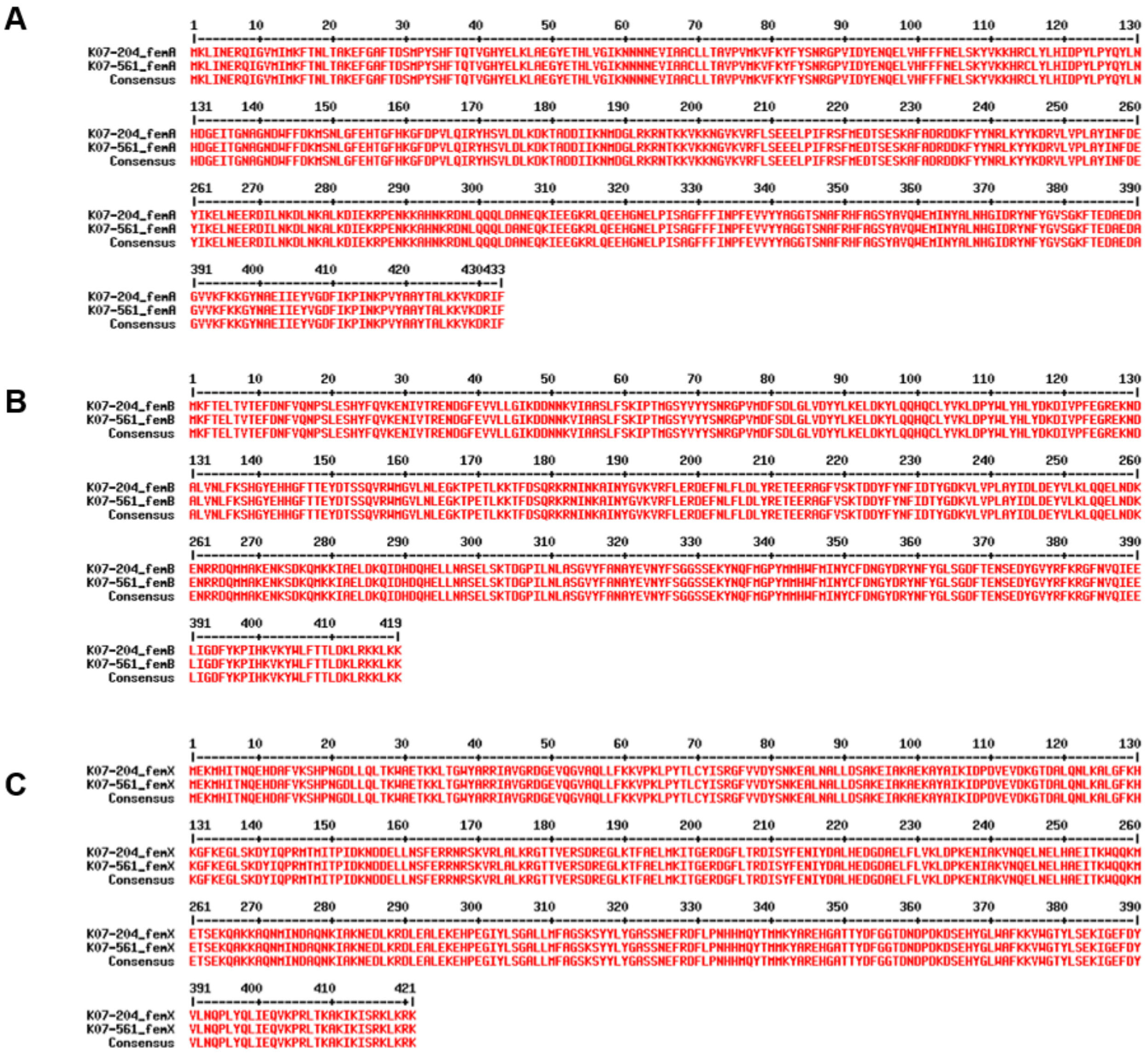

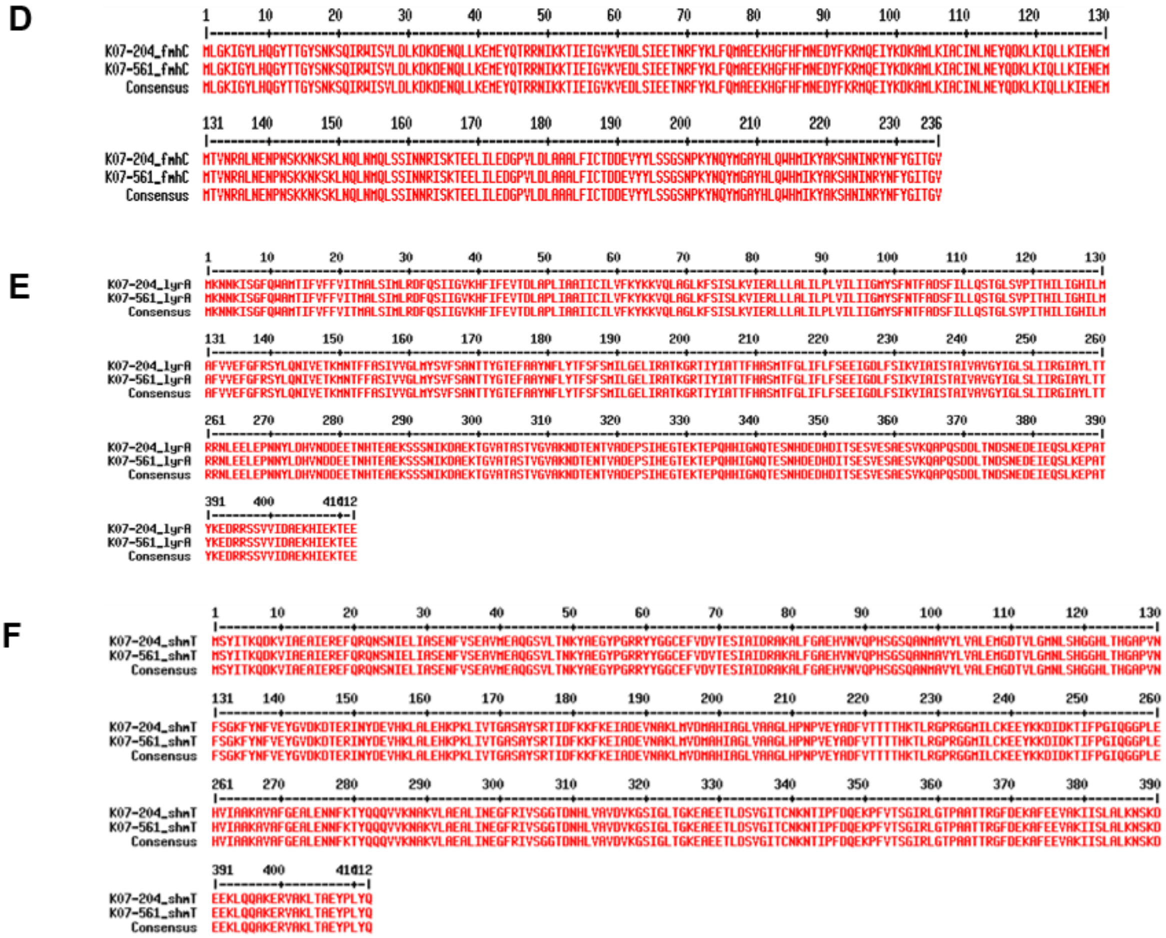

Supplementary Fig. S5. Cloning, sequencing, multiple sequence alignment to assess the mutation(s) upon translated DNA sequences. (A-F) Cloning of **(A)** *femA*, **(B)** *femB*, **(C)** *femX*, **(D)** *fmhC*, **(E)** *lyrA* **(F)** and *shmT* into pCR2.1TOPO cloning vector followed by DNA sequencing of multiple clones to assess mutation, if any, responsible for differential lysostaphin resistance between human isolates of ST72 K07-204 (lys^r) and K07-561 (lys^s). The sequenced DNA were translated *in-silico* to get amino acid sequences. The amino acid sequences of lysostaphin resistant (lys^r) K07-204 and lysostaphin susceptible (lys^s) K07-561 showed 100% identity. These results indicated that no known mechanism exists to explain the differential lysostaphin resistance between the lysostaphin resistant (lys^r) K07-204 and lysostaphin susceptible (lys^s) K07-561, human isolates of ST72.

Supporting Fig. S6

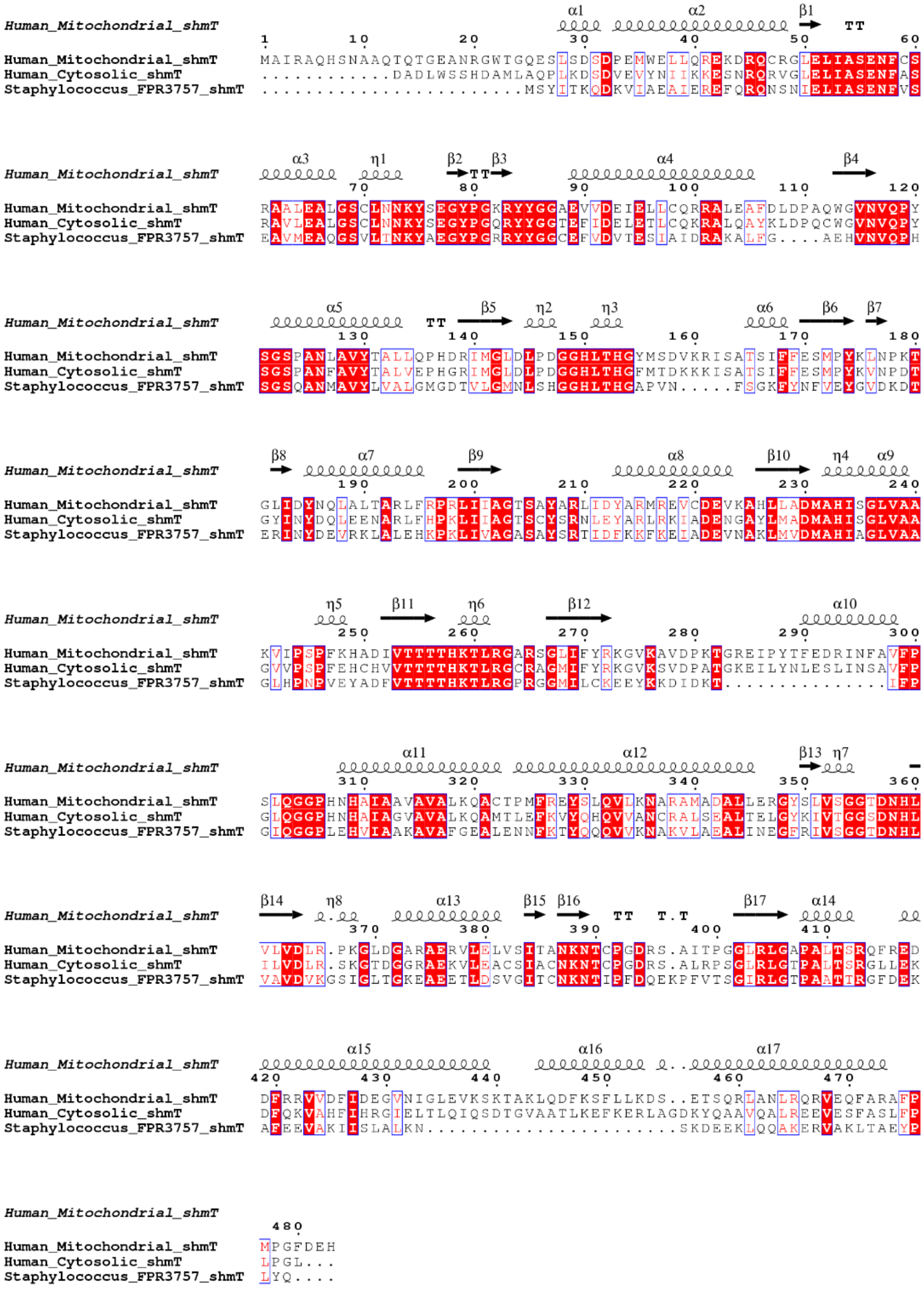

Supporting Fig. S6. Alignment of SHMT from *S. aureus* USA300 with human *SHMTs* to assess the overall similarity and identity. Alignment results showed a significantly high identity with two humans SHMTs, human cytosolic (UniProtKB - P34896) and mitochondrial SHMT (UniProtKB - P34897) with SHMT of *S. aureus* USA300 FPR3757 (CP000255.1). The human cytosolic and mitochondrial SHMT displayed 63.45% identity, while the human cytosolic and mitochondrial SHMT displayed 45.5%and 42% identity with SHMT of *S. aureus* USA300 FPR3757, respectively

Supporting Fig. S7

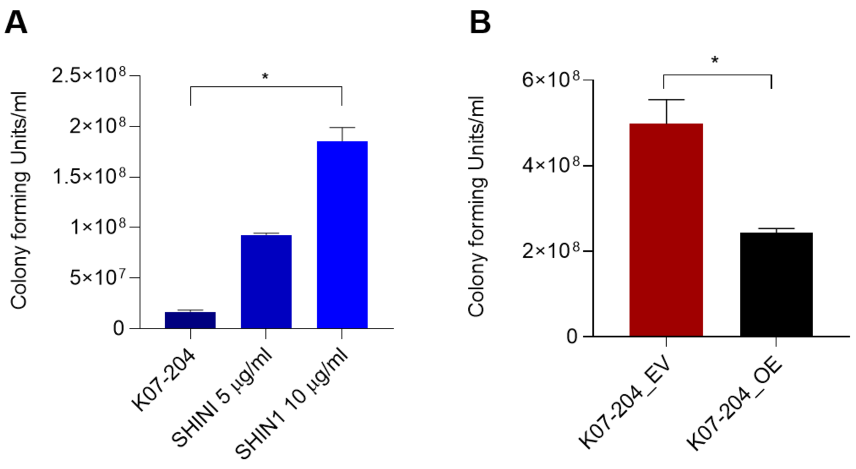

Supporting Fig S7. Role of SHMT in lysostaphin resistance of K07-204 human isolate of ST72. (**A**) The phenotypic assessment of lysostaphin resistance/susceptibility of K07-204 upon SHIN1-mediated inhibition of SHMT wherein the inhibition of SHMT marginally enhanced the resistance of K07-204, while (**B**) the overexpression of *shmT* (K07-204 with pRMC2_*shmT*) showed reduced lysostaphin resistance of K07-204. The lysostaphin killing assay was performed by using 5 units for 10 min incubation.

Supporting Fig. S8

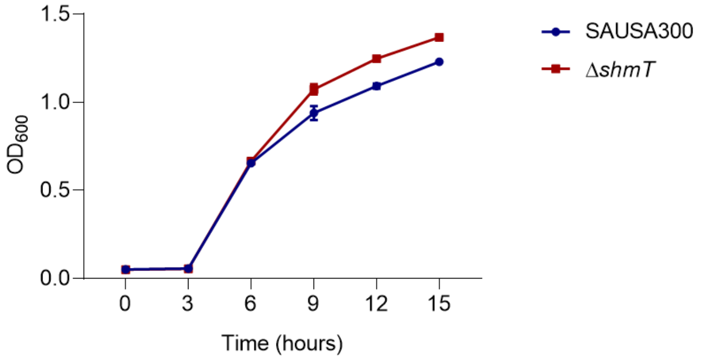

Supporting Fig. S8. The role of *shmT* on the fitness of *S. aureus* USA300. The role of *shmT* on the fitness of SAUSA300 was assessed by comparing the growth of wild type SAUSA300 and Δ*shmT* knockout in TSB media for 16h. The growth of the Δ*shmT* knockout and wild type SAUSA300 was found to be comparable.

Supporting Fig. S9

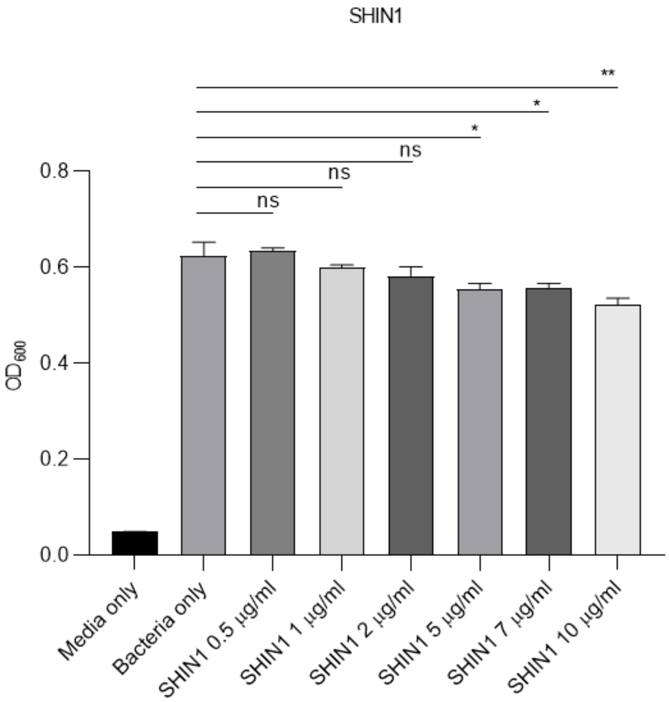

Supporting Fig. S9. Serine hydroxymethyltransferase inhibitor 1 (SHIN1) toxicity to *S. aureus* USA300 cells at varying concentrations. The SHIN1 showed insignificant inhibition of bacterial growth up to 2 µg/mL while a mild inhibition in the cell division was observed beyond 2 to 10 µg/mL, measured by estimating the inhibition of cell density at $OD_{600\ nm}$.

Table S1. *S. aureus* ST72 isolates

Sequence type 72	MRSA/MSSA	Source of Isolation	Reference
K01-140	MRSA	Human	[1]
K07-204	MRSA	Human	
K07-322	MRSA	Human	
K01-799	MSSA	Human	
K07-006	MSSA	Human	
K07-561	MSSA	Human	
05-B-52	MRSA	Animal	
05-B-60	MRSA	Animal	
08-B-93	MRSA	Animal	
08-P-236	MRSA	Animal	
4-009	MRSA	Soil	

Table S2. Primers used in the study

Purpose	Name	Sequence (5'-3')	Reference/Source	Amplicon size (bp)
PCR	*lss*_fwd	GCTATTGGACTGAGTACATTTGCC	This study	Not amplified
	*lss*_rev	CTGCGGCATGCTTCTAAATGGACCAGTC		
	*epr*_fwd	CTAYWCACATMGMGGTCCWGTCATRRAC	This study	Not amplified
	*epr*_rev	TTAGAATTAGGGTTTTCTTTTAAT		
	*fmhC*_fwd_*KpnI*	AACATAGGTACCATGAAATTTTCAACTTTAAGTG	This study	1245
	*fmhC*_rev_*EcoRI*	AAGATAGAATTCTCAAACCTTATAAATAAGTTTTGC		
	*femA*_fwd_*KpnI*	AACATAGGTACCTTGCAGAGGGGAAATAGAAAAACTGC	This study	1338
	*femA*_rev_*EcoRI*	AAGATAGAATTCCTAAAAAATTCTGTCTTTAACTTTTT		
	*femB*_fwd_*KpnI*	AACATAGGTACCATGAAATTTACAGAGTTAACTG	This study	1260
	*femB*_rev_*EcoRI*	AAGATAGAATTCCTATTTCTTTAATTTTTTACGT		
	*femX*_fwd_*KpnI*	AACATAGGTACCATGGAAAAGATGCATATCACTAATC	This study	1266
	*femX*_rev_*EcoRI*	AAGATAGAATTCCTATTTTCGTTTTAATTTACGAG		
	*lyrA*_fwd_*KpnI*	AACATAGGTACCATGAAGAACAATAAAATTTCTG	This study	1260
	*lyrA*_rev_*EcoRI*	AAGATAGAATTCTTATTTGTTTTTATCTGAAGATTG		
	*shmT*_fwd_*KpnI*	AACATAGGTACCATGTCTTATATCACCAAGCAAG	This study	1239
	*shmT*_rev_*EcoRI*	AAGATAGAATTCTTATTGATATAGAGGATATTCAGC		
qRT-PCR	*gyrA*_fwd	CGTCAACGTATTGTTGTCAC	This study	180
	*gyrA*_rev	ACACTAGCATTTGCATCCTT		
	*shmT*_fwd	TCGGAAGCGGTTATGGAA	This study	196
	*shmT*_rev	CAGCCATGTTCGCTTGTG		

Table S3. Staphylococcal strains/isolates and plasmid used in the study

Strains	Organisms	Descriptions	Reference/Source
Strains	*Escherichia coli* DH5α	*F- endA1 glnV44 thi-1 recA1 relA1 gyrA96 deoRnupG Φ80dlacZΔM15 Δ(lacZYA-argF) U169, hsdR17(rK- mK+), λ–*	Invitrogen, USA
	E. coli DH5α_pRMC2	For amplification of pRMC2 vector, amp^r	This study
	WT USA300 FPR3757	JE2, wild-type epidemic community-associated methicillin-resistant *S. aureus* isolate USA300 LAC	NARSA
	RN4220	Restriction-deficient strain of NCTC8325	**[2]**
	WT *Staphylococcus simulans*	Lysostaphin synthesizing (*lss*) and resistance gene (*epr*)	Lab collection
	WT *Staphylococcus saprophyticus*	Lysostaphin resistant strain	KCTC3345
	RN4220_pRMC2	For amplification of pRMC2 vector in *S. aureus* RN4220 as cloning intermediate, Cm^r	This study
	RN4220_pRMC2_*shmT*	For amplification of pRMC2_*shmT* in *S. aureus* RN4220 as cloning intermediate, Cm^r	
	Δ*shmT*	Knock out of *shmT* gene, Em^r	Nebraska library
	SAUSA300_pRMC2	SAUSA300_EV, Cm^r	This study
	Δ*shmT*_pRMC2	Δ*shmT*_EV, Cm^r	This study
	Δ*shmT*_pRMC2_*shmT*	Δ*shmT*_Comp, Cm^r	This study
	SAUSA300_pRMC2_*shmT*	SAUSA300_OE, Cm^r	This study
	E. coli DH5α_pCR2.1 TOPO_ *K07-561_fmhC*	P_{lac}, *K07-561_fmhC*, Km^r, Amp^r	This study
	E. coli DH5α_pCR2.1 TOPO_*K07-561_femA*	P_{lac}, *K07-561_femA*, Km^r, Amp^r	This study
	E. coli DH5α_pCR2.1 TOPO_*K07-561_femB*	P_{lac}, *K07-561_femB*, Km^r, Amp^r	This study
	E. coli DH5α_pCR2.1 TOPO_*K07-561_femX*	P_{lac}, *K07-561_femX*, Km^r, Amp^r	This study
	E. coli DH5α_pCR2.1 TOPO_*K07-561_lyrA*	P_{lac}, *K07-561_lyrA*, Km^r, Amp^r	This study
	pCR2.1 TOPO_ *K07-561_shmT*	P_{lac}, *K07-561_shmT*, Km^r, Amp^r	This study

	E. coli DH5α_pCR2.1 TOPO_*K07-204_fmhC*	P_{lac}, *K07-204_fmhC*, Km^r, Amp^r	[3]
	E. coli DH5α_pCR2.1 TOPO_*K07-204_femA*	P_{lac}, *K07-204_ femA*, Km^r, Amp^r	Invitrogen
	E. coli DH5α_pCR2.1 TOPO_*K07-204_femB*	P_{lac}, *K07-204_femB*, Km^r, Amp^r	
	E. coli DH5α_pCR2.1 TOPO_*K07-204_femX*	P_{lac}, *K07-204_femX*, Km^r, Amp^r	
	E. coli DH5α_pCR2.1 TOPO_*K07-204_lyrA*	P_{lac}, *K07-204_lyrA*, Km^r, Amp^r	
	pCR2.1 TOPO_*K07-204_shmT*	P_{lac}, *K07-204_shmT*, Km^r, Amp^r	
Native Plasmids	pRMC2	Expression vector under control of tetracycline inducible P*xyl*/*tetO*, Amp^r, Cm^r	
	pCR2.1 TOPO	Expression vector under control of lactose inducible promoter P_{lac}, Km^r, Amp^r	

References

1. Ko, K. S.; Lim, S. K.; Jung, S. C.; Yoon, J. M.; Choi, J. Y.; Song, J. H., Sequence type 72 meticillin-resistant *Staphylococcus aureus* isolates from humans, raw meat and soil in South Korea. *J Med Microbiol* **2011,** 60, (Pt 4), 442-5.

2. Peng, H. L.; Novick, R. P.; Kreiswirth, B.; Kornblum, J.; Schlievert, P., Cloning, characterization, and sequencing of an accessory gene regulator (*agr*) in *Staphylococcus aureus*. *J Bacteriol* **1988,** 170, (9), 4365-72.

3. Corrigan, R. M.; Foster, T. J., An improved tetracycline-inducible expression vector for *Staphylococcus aureus*. *Plasmid* **2009,** 61, (2), 126-9.
